# Age-associated increase of the active zone protein Bruchpilot within the honeybee mushroom body

**DOI:** 10.1371/journal.pone.0175894

**Published:** 2017-04-24

**Authors:** Katrin B. Gehring, Karin Heufelder, Harald Depner, Isabella Kersting, Stephan J. Sigrist, Dorothea Eisenhardt

**Affiliations:** 1 Freie Universität Berlin, Institut für Biologie—Neurobiologie, Berlin, Germany; 2 Freie Universität Berlin, Institut für Biologie–Genetik, Takustraße 6, Berlin, Germany; University of Cologne, GERMANY

## Abstract

In honeybees, age-associated structural modifications can be observed in the mushroom bodies. Prominent examples are the synaptic complexes (microglomeruli, MG) in the mushroom body calyces, which were shown to alter their size and density with age. It is not known whether the amount of intracellular synaptic proteins in the MG is altered as well. The presynaptic protein Bruchpilot (BRP) is localized at active zones and is involved in regulating the probability of neurotransmitter release in the fruit fly, *Drosophila melanogaster*. Here, we explored the localization of the honeybee BRP (*Apis mellifera* BRP, AmBRP) in the bee brain and examined age-related changes in the AmBRP abundance in the central bee brain and in microglomeruli of the mushroom body calyces. We report predominant AmBRP localization near the membrane of presynaptic boutons within the mushroom body MG. The relative amount of AmBRP was increased in the central brain of two-week old bees whereas the amount of Synapsin, another presynaptic protein involved in the regulation of neurotransmitter release, shows an increase during the first two weeks followed by a decrease. In addition, we demonstrate an age-associated modulation of AmBRP located near the membrane of presynaptic boutons within MG located in mushroom body calyces where sensory input is conveyed to mushroom body intrinsic neurons.

We discuss that the observed age-associated AmBRP modulation might be related to maturation processes or to homeostatic mechanisms that might help to maintain synaptic functionality in old animals.

## Introduction

Chemical synapses are responsible for signal transmission in the nervous system of animals. Their pre- and postsynaptic structures are altered during aging and these alterations are thought to underlie an age-dependent decline of cognitive function [1, 2]. Age-dependent synaptic alterations can be observed in presynaptic terminals as well as in postsynaptic spines of both vertebrates and invertebrates [3] and might be a consequence of evolving long-lived neurons that underlie long-term memories and thus complex behavior [4]. Accordingly, all animals that have the ability to form long-term memories and display complex behavior might show neuronal aging.

One invertebrate animal that has the ability to form long-term memories, displays rather complex behaviors, such as navigation, dance communication and social behavior based on learning and memory formation [5], and that shows age-associated alterations of central nervous system is the honeybee *(Apis mellifera)*. Honeybee mushroom bodies (MBs), prominent brain neuropils involved in processing sensory information, have been shown to be modified in an age-associated manner [6–10]. Each MB, one per hemisphere, consists of two cup-shaped calyces that can be further divided into lip (receiving olfactory input), collar (receiving mainly visual input) and basal ring (receiving multimodal input) [11–15]. All three input regions contain numerous synaptic complexes called microglomeruli (MG), which consist of a presynaptic bouton, formed by a sensory projection neuron, surrounded by a basket of numerous tiny Kenyon cell dendrites [16–19]. Each presynaptic bouton has, depending on the MB sensory input region, about 40 to 70 active zones where neurotransmitter release takes place [8].

In the MB sensory input regions, density and volume of the MG as well as alterations on the level of individual synapses are associated with age. The packing density of presynaptic boutons decreases with age in lip and dense collar resulting in fewer boutons in a defined area [8–10]. However, at the same time projection neuron boutons in MB lip and collar of 35-day-old bees have a larger individual size, an increased number of ribbon synapses, more postsynaptic contacts per active zone, and, in the collar, an increased number of active zones per bouton compared with 1-day-old bees [8]. These age-dependent structural changes in MB calyxes might result in an increased neurotransmitter release [8]. One precondition for this would be an increase in the abundance of presynaptic proteins that are involved in the transmitter release of presynapses in MG. One of these presynaptic proteins involved in transmitter release is Bruchpilot (BRP), found at the cytomatrix at the active zone in most if not all presynapses of various insects [20, 21]. The N-terminus of DmBRP shows sequence homology to the vertebrate CAST/ERC (CAST = cytomatrix at the AZ associated structural protein, ERC = ELKS-Rab6-interacting protein CAST) and its C-terminus has a large domain similar to cytoskeletal proteins such as Plectin, Myosin heavy chain and Restin [20]. In fruit flies, antibodies against DmBRP specifically label presynaptic active zones [20, 22]. Reducing the level of *Drosophila melanogaster* BRP (DmBRP) results in decreased neurotransmitter release and mislocalized Ca^2+^ channels [20, 22, 23]. This led to the hypothesis that DmBRP is involved in concentrating synaptic vesicles close to Ca^2+^ channels, and thus in the regulation of synaptic transmission [22–24]. In fact, recent work using an optical sensor that allowed for single active zone resolution showed that local levels of DmBRP scale directly with the likelihood of evoked release as revealed by *in vivo* analysis of larval *Drosophila* neuromuscular junctions [25, 26].

Up to date, nothing is known about *Apis mellifera* BRP (AmBRP) in the honeybee brain. We here asked where AmBRP is localized in the MG of the honeybee MB sensory input regions (i.e. lip and collar of the calyces), and whether AmBRP expression is modified in an age-associated manner in honeybee MBs.

## Material and methods

### Animals and cohort experiment

For the characterization of the BRP antibodies, worker honeybees (*Apis mellifera*) were caught in front of their hives in the afternoon at the Freie Universität Berlin, immobilized by cooling, and harnessed in small plastic tubes.

For experiments exploring AmBRP levels during aging we utilized age-matched cohorts of summer honeybees (*Apis mellifera*) [27]. A brood comb of a single colony located at the Freie Universität Berlin was caged shortly before bees were due to eclose, wrapped into aluminum foil to prevent the escape of newly emerged bees into the hive, and placed back into the natal colony. Twenty four hours later, the newly eclosed bees were collected from the brood comb and were either immediately processed for Western blotting or immunohistochemical procedures (1-day-old bees), or marked with a colored spot (acrylic paint, Hobby Line, C. Kreul GmbH & Co.KG, Hallerndorf, Germany) on the dorsal part of the thorax and transferred back into the natal colony. Marked bees were collected from inside the hive between 10:00 and 14:00 at age 8, 15, 29 and 43 d, regardless of the task they were performing. Cohort experiments were conducted from May to September in 2012.

Wild type *Drosophila melanogaster* (Canton S, Berlin, 2–3 days old) used for Western blotting experiments were raised on a standard cornmeal-molasses-agar medium on a 12-/12-hour light and dark cycle at 25°C with 60% humidity.

### Antibody characterization: BRP^last200^

**Fig 1:**
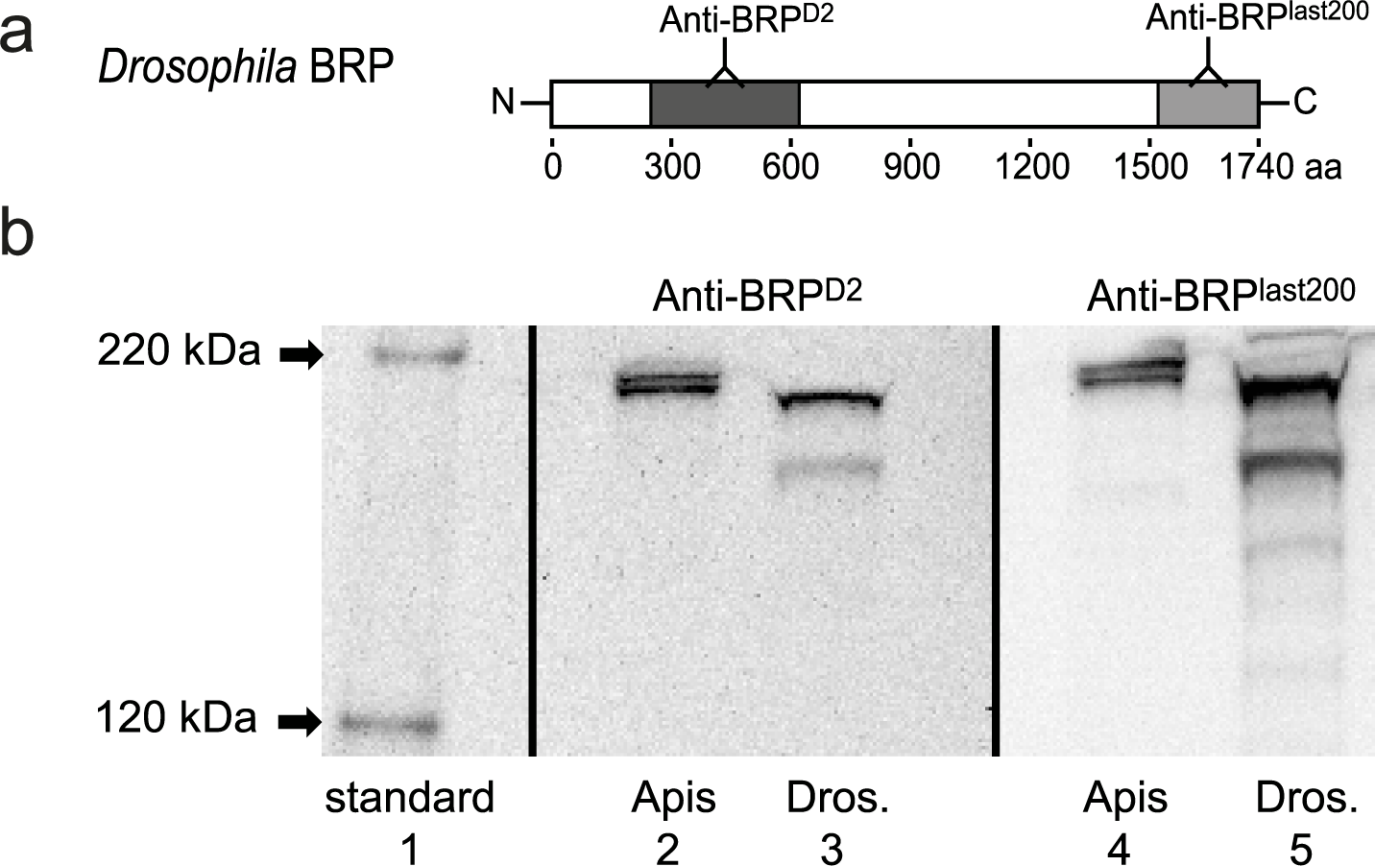
AmBRP variants detected in the honeybee brain. **a** Map of the BRP^last200^ and the BRP^D2^ antibodies’ epitopes in the large *Drosophila* BRP isoforms D (NP_724796). **b** Immunoblot of honeybee central brain and fruit fly head homogenate. The BRP^last200^ antibody and the BRP^D2^ antibody recognize two bands around 220 kDa in honeybee central brains (lane 2, 4), and two major bands and several light bands between 120 and 220 kDa in fruit fly heads (lane 3, 5).

The rabbit polyclonal BRP^last200^ antibody [28] (made by the Sigrist lab, Freie Universität Berlin, Berlin, Germany), was raised against the last 200 amino acids of the large *Drosophila melanogaster* Bruchpilot isoforms (e.g. isoform D, NP_724796). In brief, the corresponding coding sequence was amplified from the bruchpilot cDNA [20] and cloned into bacterial expression vectors pGEX-6P-1 and pET28a using BamHI and XhoI restriction sites. The purified GST-fusion protein was injected into a rabbit and the antisera were separately affinity purified with the 6x-His-BRP-last200aa fusion protein. This antibody was used as primary antibody to detect BRP in Western blot and immunohistochemistry (BRP^last200^) (Table 1). In a BLAST analysis, we explored the homology between the last 200 amino acids of *Drosophila melanogaster* BRP proteins (isoform D, accession number NP_724796) and *Apis mellifera* homologous BRP proteins (NP_001242968.1, XP_006561572.1, XP_006561571.1) and ensured that no other protein containing the epitope of the BRP^last200^ antibody was found in the appropriate honeybee databases (http://www.ncbi.nlm.nih.gov/mapview/map_search.cgi?taxid=7460). To demonstrate that the BRP^last200^ antibody labels an antigen of an appropriate size in *Apis mellifera* (Fig 1A), we used Western blotting (Fig 1B). In addition, we compared honeybee and fly homogenate on Western blot using the BRP^last200^ antibody and another already published antibody binding to a different region in the DmBRP protein: anti-BRP^D2^ [20, 22, 23] (Fig 1A).

**Table 1:** Primary antibodies used in this study.

Name of primary antibody	Immunogen	Manufacturer, Cat. No., Species raised in, Mono-/Polyclonal	Antibody dilution
Anti-BRP^last200^	Last 200 amino acids of large *D*. *melanogaster* Bruchpilot isoforms (e.g. amino acids 1540–1740 of isoform D, protein accession number: NP_724796)	Prof. Dr. Sigrist, rabbit, polyclonal (Ullrich et al., 2015)	Western blot: 1:6,000 Immuno-histochemistry: 1:500
Anti-BRP^D2^	*D*. *melanogaster* Bruchpilot domain D2 (amino acids 268–617)	Prof. Dr. Sigrist, rabbit, polyclonal (Fouquet et al., 2009)	Western blot: 1:20,000
Anti-α-Tubulin mouse mAb (DM1A)	Native chicken brain microtubules	Calbiochem, Sandhausen, Germany, #CP06, RRID: AB_212802, mouse, monoclonal	Western blot: 1:3,000
Anti-SYNORF1	*D*. *melanogaster* synapsin Glutathione S-transferase fusion protein, epitope: 341 LFGGMEVCGL 350	DSHB, Iowa City, IA, USA, Anti-SYNORF1, #3C11, RRID: AB_2313867, mouse, monoclonal	Western blot: 1:1,500 Immuno-histochemistry: 1:100

### Characterization of tissue markers

Phalloidin is a toxin that binds to neuronal F-actin, which is enriched in the spines of Kenyon cell dendrites in bees [29, 30]. Therefore we used Alexa Fluor 546 Phalloidin (0.3 units, Invitrogen, Carlsbad, CA, USA) to label postsynaptic spines of Kenyon cells. For immunolabeling of Synapsin, we used a monoclonal mouse antibody against the *Drosophila* synaptic vesicle-associated protein Synapsin I (anti-SYNORF1, gift from Prof. Dr. Erich Buchner, Würzburg, Germany, now #3C11, RRID: AB_2313867, in the Developmental Studies Hybridoma Bank, Iowa City, IA, USA). The SYNORF1 antibody was raised against the fusion protein *Drosophila melanogaster* Synapsin and Glutathione S-transferase [31]. The epitope (aa 341 LFGGMEVCGL 350) has been described [32]. Previous publications (8, 10, 30, 33) demonstrate that the SNYORF1 antibody labels presynaptic terminals in the honeybee MBs. As loading control in Western blots we used α-Tubulin [33], which was detected with a monoclonal mouse α-Tubulin antibody (#CP06, RRID: AB_212802, Calbiochem, Sandhausen, Germany). Secondary antibodies directed against mouse or rabbit IgG coupled to horseradish peroxidase (1:10,000 for anti-mouse IgG and 1:5,000–1:2,000 for anti-rabbit IgG, Sigma-Aldrich, St. Louis, MO, USA) were used in Western blots, Cy5-conjugated goat anti-rabbit and Alexa 488-conjugated goat anti-mouse secondary antibody (both 1:200, Jackson ImmunoResearch Laboratories, Cambridgeshire, UK) in tissue staining (immunohistochemistry).

### Western blotting

First, bees were anesthetized on ice, then a window was cut into the head capsule, and trachea and glands were removed. For Western blot characterization of BRP antibodies, dissected central bee brains (excluding optical lobes) were homogenized for 30 s in 2x Laemmli Sample Buffer (BioRad, München, Germany) containing 0.10 M DTT (30 μl per bee brain). Central bee brains were treated as described in Gehring et al. (2016) [27]. Flies were frozen in liquid nitrogen and vortexed to separate head and the rest of the body. Ten fly heads were homogenized for 30 s in 30 μl 2x Laemmli Sample Buffer containing 0.10 M DTT. Samples were subjected to sodium dodecylsulfate-polyacrylamide gel electrophoresis (SDS-PAGE) using gradient gels (Mini-PROTEAN TGX Precast Gels 4–15%, BioRad, München, Germany) and transferred onto PVDF membrane (BioRad, München, Germany). Membranes were cut at the level of the ~ 70 kDa. As molecular weight markers, we used Magic Mark Standard (LC5602, Thermo Fisher scientific, Waltham, MA) or peqGOLD protein marker VII (Peqlab, Erlangen, Germany). Blocking, incubation with primary and secondary antibodies and detection of signal intensities was done as described in Gehring et al. (2016) [27]. As primary antibodies we used: anti-BRB^last200^, anti-BRP^D2^, anti-SYNORF1 and, as standard, anti-α-Tubulin (Table 1). Chemiluminescence signals were captured with a LAS1000 camera and the software Image Reader LAS-1000 2.60 (Fujifilm Corporation, Tokyo, Japan). Signal intensities were quantified with MultiGauge version 3.0 (Fujifilm Corporation, Tokyo, Japan). The acquired value of the bands detected with a specific antibody (anti-BRB^last200^, anti-SYNORF1, anti-α-Tubulin) was normalized to the mean value of all bands detected with this antibody on the same blot. The two main AmBRP and α-Tubulin bands were measured as one band. Finally, the ratio between the normalized AmBRP and α-Tubulin values or the normalized Synapsin and α-Tubulin values was calculated for each sample. One sample represented one bee brain.

### Immunohistochemistry

Immunohistochemistry was done as described in Gehring et al. (2016) [27]. Brain slices were incubated with anti-BRB^last200^ and anti-SYNORF1 to label AmBRP and Synapsin, respectively. Alexa Fluor 546 Phalloidin was used to label neuronal f-actin.

### Confocal microscopy, image processing and data acquisition

Labeled brain slices were scanned with a confocal laser-scanning microscope (TCS SP2, Leica Microsystems, Wetzlar, Germany) equipped with an argon laser (488 nm), a helium-neon laser (633 nm) and a green helium-neon laser (543 nm). Optical sections were taken at a format of 1024 x 1024 pixels using a 10x air objective (HC PL APO, NA/0.4, Leica Microsystems, Wetzlar, Germany) for overview images and a 63x oil-immersion objective (HCX PL APO, NA/1.32, Leica Microsystems, Wetzlar, Germany) for images with higher magnification. Channels of triple-labeled preparations were merged with the use of pseudocolors using Zeiss LSM Image Browser (Version 3.2.0.115, Carl Zeiss Microscopy GmbH, Jena, Germany). As the staining patterns were similar in different preparations of the same experimental group, figures show representative examples.

For analyzing anti-BRP^last200^ and anti-SYNORF1 signals in the MBs, we chose one slice per bee where the central complex and the medial lobes of the MBs were present to ensure scanning of similar regions in each bee brain. Optical sections were taken in one optical plane from both medial MB calyces with the 63x objective. Microscope and scanning settings were kept identical for comparative brain slices of other bees. Digital images were further processed and quantified using ImageJ (ImageJ version 1.46m, Wayne Rasband, National Institutes of Health, USA, http://imagej.nih.gov/ij).

To measure anti-BRP^last200^ and anti-SYNORF1 signals, we placed two regions of interest (ROIs) in the dense collar and two ROIs in the lip region of both medial calyces in the scanned images. Each ROI covers a square area of 400 μm^2^ (86 x 86 pixels, pixel size 232 nm). The signal intensity of each pixel per ROI was measured separately for the anti-BRP^last200^ and the anti-SYNORF1 staining, i.e. for each channel. Next, the lowest intensity value of a channel measured per ROI was subtracted from all other values in this ROI. Each of these background corrected values was normalized to the highest calculated value in its ROI to obtain relative values between zero and one. To detect those pixels indicating the presence of an antibody staining, each relative value that was higher than an antibody- and region-specific threshold was defined as signal (anti-BRP^last200^-positive or anti-SYNORF1-positive pixels). This threshold was defined as the sum of a constant, antibody- or dye-specific value (0.1 for Alexa Fluor 546 Phalloidin, 0.05 for anti-SYNORF1, and 0.15 for anti-BRP^last200^, constant values were set by the experimenter) and the median of all values in one ROI. In doing so, potential discrepancies in value intensities between different ROIs based on the staining method and between sample differences were taken into account. For the quantification of anti-SYNORF1- and anti-BRP^last200^-positive pixels in lip and collar of 1-, 8-, 15-, 29- and 43-day-old bees, we calculated the median number of anti-BRP^last200^- and anti-SYNORF1-positive pixels per ROI for collar and lip of each bee and the ratio between the two medians.

### Statistical analysis

The values from quantitative Western blot analyses and from the quantification of antibody signal intensities were in most cases non-normally distributed. Therefore groups were tested with the Kruskal-Wallis test and the two-tailed Mann-Whitney U test for post-hoc comparisons. The alpha level was set to 0.05 and was Bonferroni-Holm corrected. All statistical analyses were performed with Statistica 12 (StatSoft, Tulsa, OK, USA).

## Results

### AmBRP variants detected in the honeybee brain

In this study, we aim to elucidate the age-associated localization of BRP in the honeybee brain. In a Western blot analysis, we examined whether BRP can be detected in honeybee brains. A BLAST analysis revealed 50–53% of identical amino acids between large DmBRP isoforms such as isoform D (NP_724796) and homologous amino acid sequences in honeybees (NP_001242968.1, XP_006561572.1, and XP_006561571.1), which we termed *Apis mellifera* BRP (AmBRP).

We utilized a polyclonal antibody generated against the last 200 amino acids of the DmBRP protein (BRP^last200^). This antibody detects two main bands of 170 kDa and 190 kDa in a Western blot analysis of wild type fly head homogenate. These two bands were also detected by a second antibody (anti-BRP^D2^) binding to the D2 domain of DmBRP [22] (Fig 1). In addition, the BRP^last200^ antibody detected three faint bands between 120 kDa and 170 kDa, most likely corresponding to other DmBRP isoforms (http://flybase.org/reports/FBgn0259246.html).

A BLAST analysis revealed 45% identical amino acids between DmBRP and AmBRP within the epitope of BRP^last200^. Moreover, no other protein containing the epitope of the BRP^last200^ antibody was found in the appropriate honeybee database, indicating that only two AmBRP isoforms can be detected with the BRP^last200^ antibody. Indeed, in bee brain homogenate, the BRP^last200^ antibody and the antibody detecting N-terminal amino acids of DmBRP (BRP^D2^), detected two bands around 220 kDa (Fig 1A and 1B) which correspond well to the calculated molecular weight of 223 kDa of a predicted 1908 amino acid sequence encoded by the *Apis mellifera* brp gene (NP_001242968). Thus, two AmBRP isoforms with a molecular weight of about 200 kDa to 220 kDa can be detected in the honeybee brain.

### AmBRP is predominantly located near the membranes of MG presynaptic boutons in MB calyces

**Fig 2:**
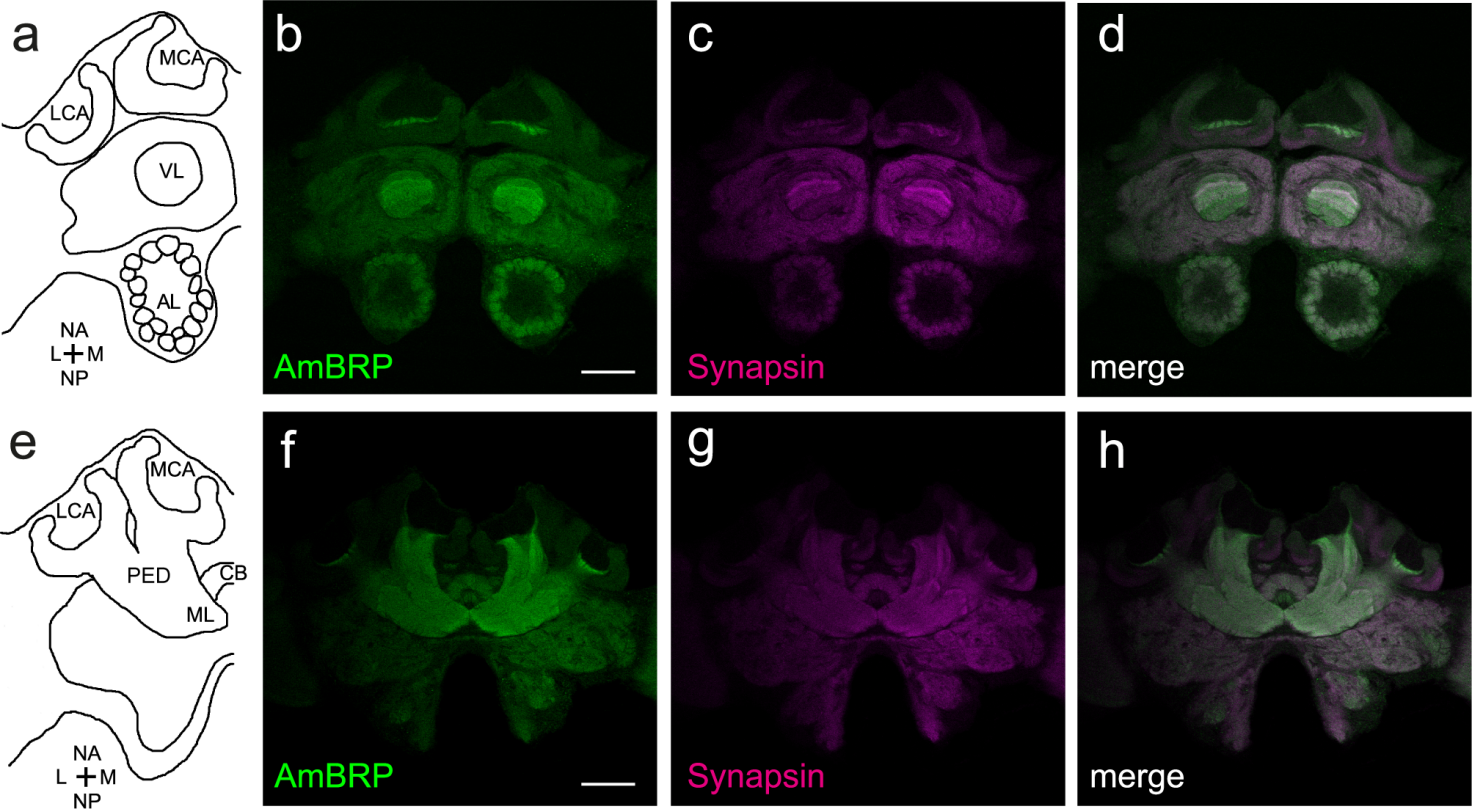
Distribution of AmBRP and Synapsin in the honeybee central brain. Optical sections of a honeybee central brain incubated with the anti-BRP^last200^ and anti-SYNORF1 to visualize the presynaptic proteins AmBRP and Synapsin. **a** Longitudinal section through a schematic honeybee brain showing ventrally located regions (nomenclature after Ito et al. (2014) [34]). **b-d** Distribution of BRP^last200^ signals (b) and anti-SYNORF1 signals (c) in ventrally located brain regions of a 29-day-old bee. Both antibodies show staining in all brain regions with almost similar distribution (d). Prominent stained regions are the vertical lobes and the antennal lobes. **e** Longitudinal section through a schematic honeybee brain showing dorsally located regions (nomenclature after Ito et al. (2014) [34]).**f-h** Distribution of BRP^last200^ signals (f) and anti-SYNORF1 signals (g) in dorsally located brain regions of a 29-day-old bee. Both antibodies show staining in all brain regions with similar distribution (h) Prominent stained regions are the peduncles, especially in the AmBRP staining. LCA, lateral calyx; MCA, medial calyx; VL, vertical lobe; PED, peduncle; ML, medial lobe; CB, central body; AL, antennal lobe; NA, neuraxis anterior; M, medial; NP, neuraxis posterior; L, lateral. Scale bars: 200 μm for b-d and f-h.

**Fig 3:**
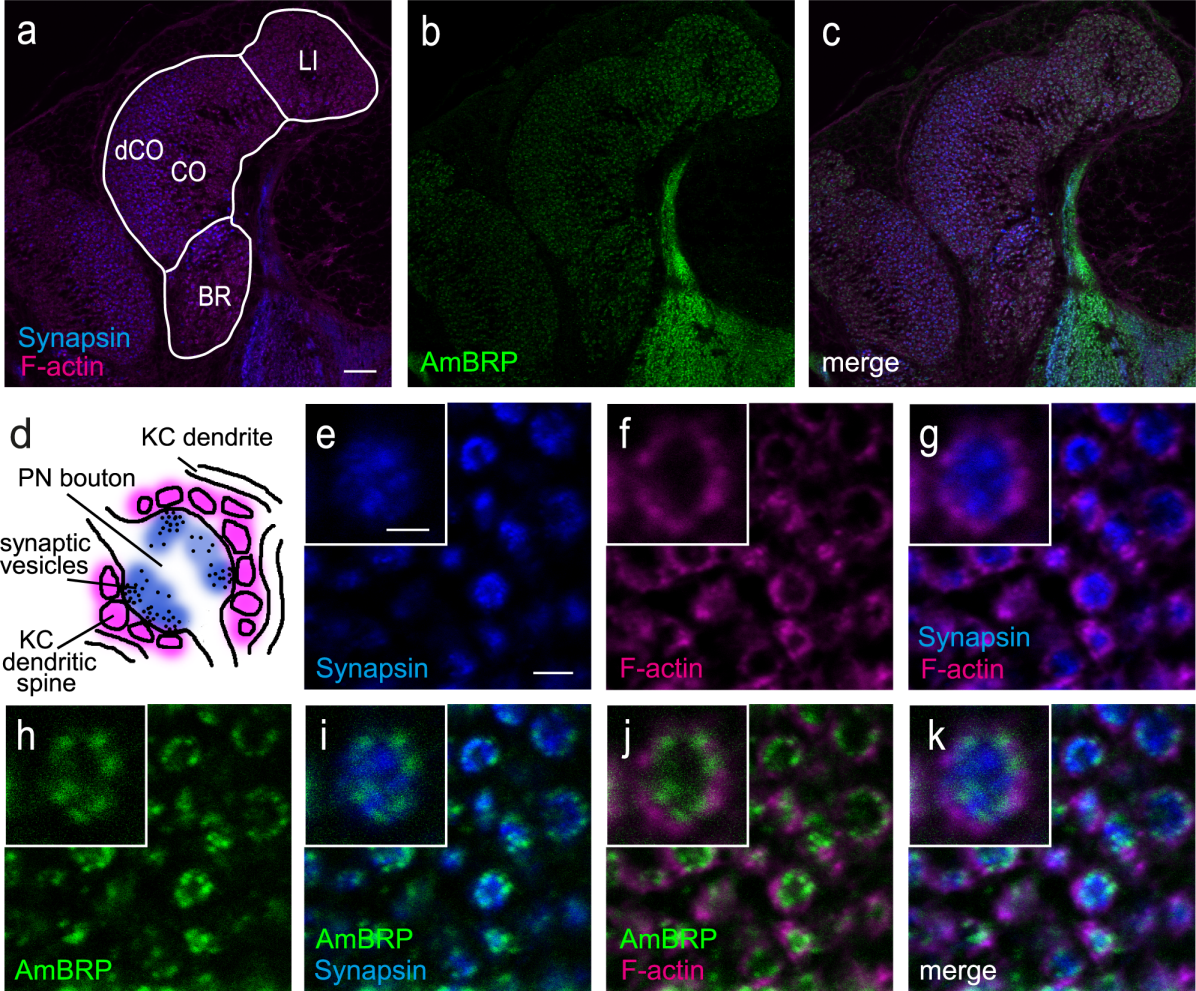
AmBRP is predominantly located in the vicinity of the membrane of presynaptic boutons within microglomeruli in the mushroom body calyces. **a-c** Confocal images of the medial calyx showing BRP^last200^ staining (AmBRP, green, b) in combination with Phalloidin staining (F-actin, magenta, a) and an anti-SYNORF1 counterstaining (Synapsin, blue, a) to visualize pre- and postsynaptic structures in a 8-day-old bee. The calyx can be subdivided into three regions, lip, collar and basal ring. Experiments focused on the lip and the dense region of the collar (dCO). **d** Schematic representation of a microglomerulus (MG) (modified after [35] showing already established pre- and postsynaptic marker (Synapsin, blue; F-actin, magenta). The bouton of a projection neuron is surrounded by spines from Kenyon cell dendrites. Anti-SYNORF1 labels the vesicle-associated protein Synapsin (blue) whereas Alexa Fluor 546 Phalloidin binds to F-actin located in dendritic spines (magenta). **e-k** Confocal images of MG in the dense collar region with labeled Synapsin (e), F-actin (f) and AmBRP (h). The F-actin signals form circles around Synapsin signals (g). AmBRP is located predominantly at the outer rim of the Synapsin-labeled signals and at the inner rim of the F-actin signals (i-k). The insets show a single, magnified MG from the corresponding image. LI, lip; CO, collar; BR, basal ring; dCO, dense collar; PN, projection neuron; KC, Kenyon cell.Scale bars: 20 μm for a-c, 2 μm for e-k, 1 μm for insets.

Next, the BRP^last200^ antibody was used for a detailed confocal microscopic analysis of AmBRP distribution in the honeybee brain (Figs 2 and 3). In addition, we labeled F-actin to visualize postsynaptic sites and Synapsin to visualize presynaptic sites [8, 10, 29, 30]. AmBRP proteins are present throughout the honeybee brain and showed synaptic localization similar to Synapsin (Fig 2). Regions predominantly stained by anti-BRP^last200^ and anti-SYNORF1 were found in the MBs, the central complex and the antennal lobes. A remarkably high signal intensity in immunolabelings of AmBRP proteins was found in the vertical lobes and the peduncles (Fig 2B–2D and 2F–2H).

In order to examine if AmBRP is localized at the membrane of presynaptic single nerve terminals (boutons) we probed its subcellular localization using pre- and postsynaptic markers. This analysis revealed that in synaptic complexes (MG) located in lip and collar of the MBs, the F-actin signals appeared in ring-like structures surrounding the anti-SYNORF1-labeled boutons (Fig 3A and 3E–3G). The anti-BRP^last200^ signals were predominantly located at the outer rim of anti-SYNORF1-labeled boutons indicating an AmBRP localization at the transition area between the anti-SYNORF1 staining and the F-actin staining, thus in areas near the presynaptic bouton membrane where synapses are formed (Fig 3H–3K).

### The levels of AmBRP and Synapsin in the central bee brain change in an age-associated manner

**Fig 4:**
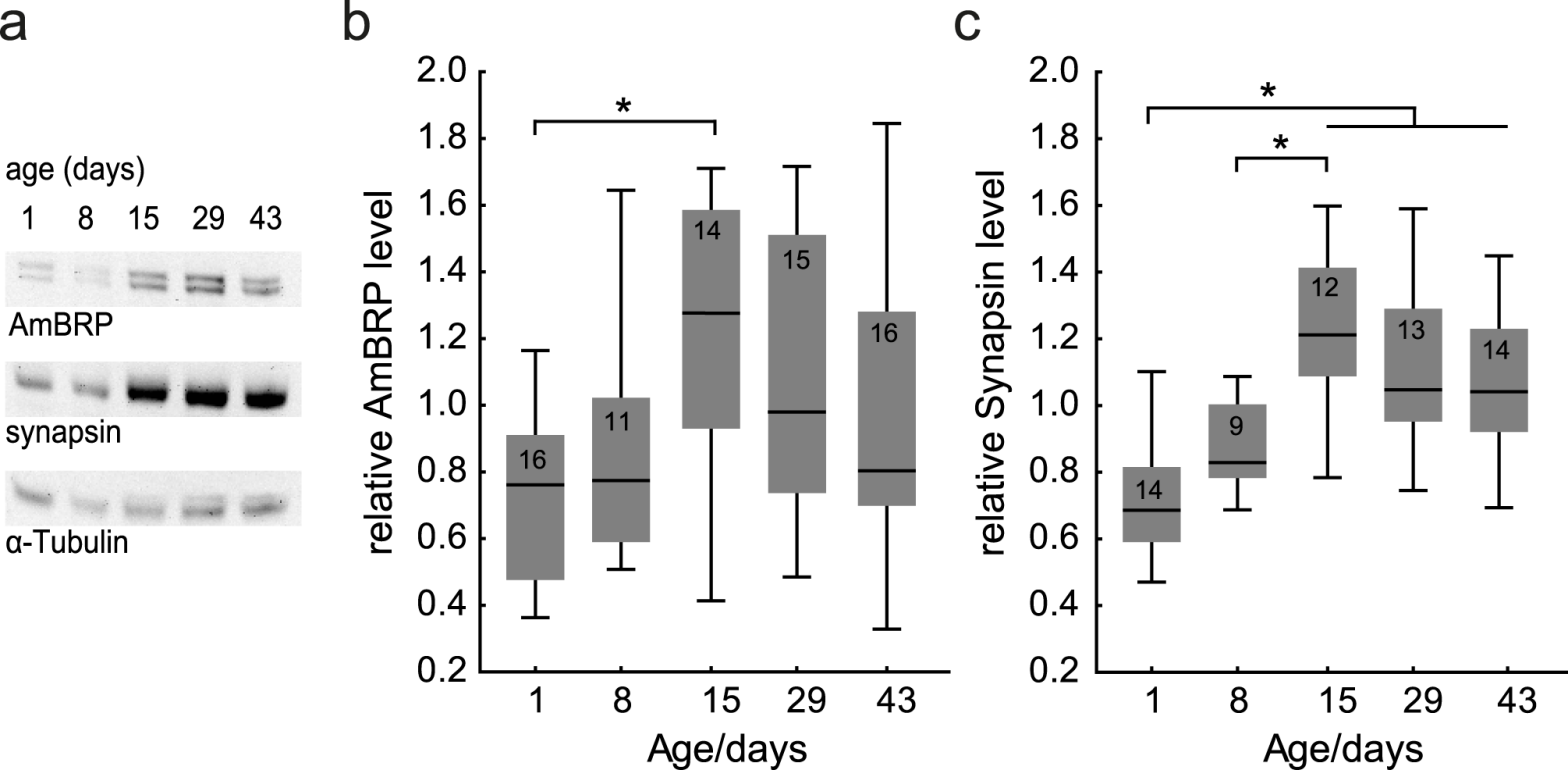
Age-associated changes in AmBRP and Synapsin levels in the central brain of *Apis mellifera*. **a** Representative Western blot of central brains from 1-, 8-, 15-, 29- and 43-day-old bees. Shown are AmBRP proteins migrating around 220 kDa, Synapsin proteins around 70 kDa and the α-Tubulin band around 60 kDa. **b-c** Results from the quantitative Western blot analysis using central brain homogenate from 1-, 8-, 15-, 29- and 43-day-old bees. **b** The level of AmBRP in the central brain is increased in the group of 15-day-old worker bees compared with 1-day-old bees. **c** The level of Synapsin is increased in the group of 15-, 29- and 43-day-old worker bees compared with 1-day-old bees. Box blots show median, 25% and 75% quartiles and value range (min-max). (*) Significant differences (p < 0.05) detected with Mann-Whitney U test after Kruskal Wallis ANOVA.

To investigate whether the level of presynaptic proteins in the bee brain is altered during aging, we quantified levels of AmBRP and Synapsin in central brains of 1-, 8-, 15-, 29- and 43-day-old bees in a Western blot analysis (Fig 4, raw data is presented in S1 Table). Of note, we found 15-day-old bees (in contrast to 8-, 29- and 43-day-old bees) to have a significantly increased AmBRP level when compared with 1-day-old bees (Kruskal-Wallis ANOVA over all groups: H_(4, N = 72)_ = 11.69, p < 0.05; Mann-Whitney U: 1 d vs. 15 d, p = 0.001) (Fig 4B). In contrast, the Synapsin level was significantly increased in 15-, 29- and 43-day-old bees compared with 1-day-old bees and differed also between 8- and 15-day-old bees (Kruskal-Wallis ANOVA over all groups: H_(4, N = 62)_ = 27.28, p < 0.001; Mann-Whitney U: 1 d vs. 15 d, p = 0.000; 1 d vs. 29 d, p = 0.000; 1 d vs. 43 d, p = 0.000; 8 d vs. 15 d, p = 0.004) (Fig 4C). These results demonstrate age-associated alterations in the level of the presynaptic proteins AmBRP and Synapsin in the honeybee central brain.

### Age-associated variation of Synapsin and AmBRP in the mushroom body lip and collar

**Fig 5:**
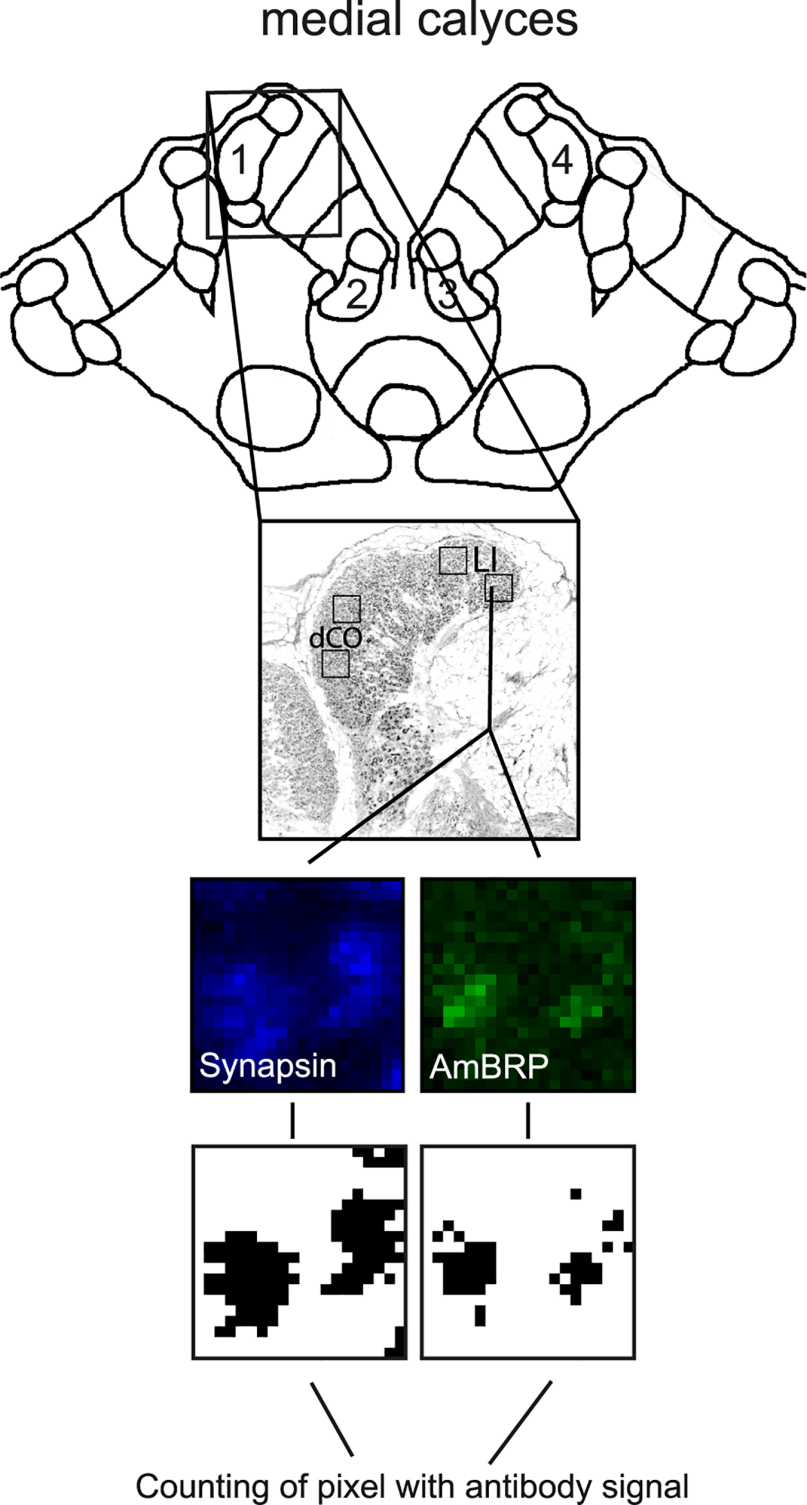
Schematic of the pixel counting method. Schema presenting the method of counting pixels with an antibody signal in regions of interest (ROIs) located in the medial calyces of the honeybee brain. Black squares in the medial calyx (F-actin labeled, scale bar is 20 μm) show the approximately location of these ROIs. Two ROIs were placed in the lip (LI) and two in the dense collar (dCO). One ROI covers an area of 400 μm^2^ (86 x 86 pixels). The number of pixels that were positive for a specific antibody (i.e. that had an intensity value over a certain threshold, for details see material and methods) were counted.

Next, we investigated how age affects the allocation of the presynaptic proteins AmBRP and Synapsin in the MB lip, an input region for olfactory information, and collar (dense collar), an input region for mainly visual information. In short, we counted the number of anti-SYNORF1- and anti-BRP^last200^-positive pixels in defined regions of interest (ROIs) in lip and collar of 1-, 8-, 15-, 29- and 43-day-old bees (see above, Fig 5). Each ROI contained a constant number of pixels (7396 pixels = 400 μm^2^). We calculated the median number of anti-BRP^last200^- and anti-SYNORF1-positive pixels per ROI and the ratio between anti-BRP^last200^- and anti-SYNORF1-positive pixels per ROI. We calculated this ratio in order to correct for age-associated variations in the presynaptic area, because anti-SYNORF1-staining is labeling presynaptic structures [8, 10, 30, 36]. The raw data of the calculated median numbers and the ratios is presented in S2 Table.

**Fig 6:**
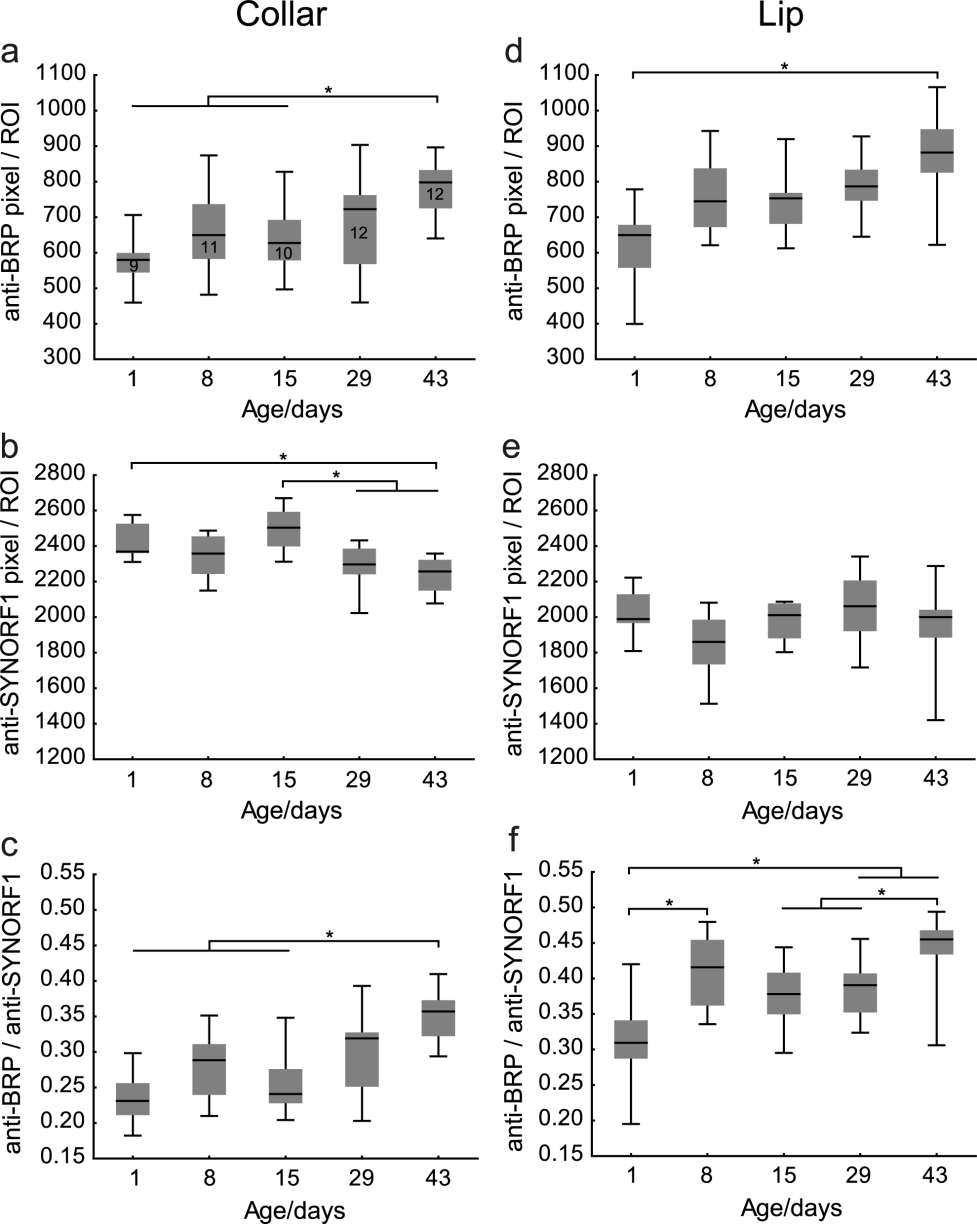
The median number of anti-BRP^last200^- and anti-SYNORF1-positive pixels per ROI varies with age in lip and collar. **a** The median number of anti-BRP^last200^-positive (anti-BRP) pixels per ROI in the collar is higher in 43-day-old bees compared with 1-, 8- and 15-day-old bees. **b** The median number of anti-SYNORF1-positive (anti-SYNORF1) pixels per ROI in the collar is lower in 43-day-old bees compared with 1- and 15-day-old bees. **c** The ratio of anti-BRP^last200^-positive pixels to anti-SYNORF1-positive pixels per ROI in the collar is higher in 43-day-old bees compared with 1-, 8- and 15-day-old bees. **d**. 1-day- and 43-day-old bees differ in their median number of anti-BRP^last200^-positive pixels per ROI in the collar. **e** The median number of anti-SYNORF1-positive pixels did not change with age in the lip. **f** The ratio of anti-BRP^last200^-positive pixels to anti-SYNORF1-positive pixels per ROI in the lip is higher in 8-day-old bees compared with 1-day-old bees and in 43-day old bees compared with 15- and 29-day-old bees. Box blots show median, 25% and 75% quartiles and value range (min-max). (*) Significant differences (p < 0.05) detected with Mann-Whitney U test after Kruskal Wallis ANOVA.

In the collar, we found that the median number of anti-BRP^last200^-positive pixels per ROI was initially rather low (less than 600 of the pixels per ROI were anti-BRP^last200^-positive) and that 43-day-old bees had significantly more anti-BRP^last200^-positive pixels per ROI than 1-, 8- and 15-day-old bees (Kruskal-Wallis ANOVA over all groups: H_(4, N = 54)_ = 17.15, p < 0.01; Mann-Whitney U: 1 d vs. 43 d, p = 0.000; 8 d vs. 43 d, p = 0.005; 15 d vs. 43 d, p = 0.005) (Fig 6A). In contrast, the median number of anti-SYNORF1-positive pixels per ROI was higher in younger bees reaching the lowest level in 43-day old bees (Kruskal-Wallis ANOVA over all groups: H_(4, N = 54)_ = 23.69, p < 0.001; Mann-Whitney U: 1 d vs. 43 d, p = 0.000; 15 d vs. 29 d, p = 0.002; 15 d vs. 43 d, p = 0.000) (Fig 6B). The ratio of anti-BRP^last200^-positive pixels to anti-SYNORF1-positive pixels per ROI was 0.23 the first day after emergence and significantly increased in 43-day-old bees compared with 1-, 8-, 15-day-old bees (median ratios are 0.23 (1 d), 0.29 (8 d), 0.24 (15 d), 0.32 (29 d), 0.36 (43 d), Kruskal-Wallis ANOVA over all groups: H_(4, N = 54)_ = 21.40, p < 0.001; Mann-Whitney U: 1 d vs. 43 d, p = 0.000; 8 d vs. 43 d, p = 0.000; 15 d vs. 43 d, p = 0.000) (Fig 6C).

In the lip, the median number of anti-BRP^last200^-positive pixels per ROI was increased in 43-day-old bees compared with 1-day-old bees (Kruskal-Wallis ANOVA over all groups: H_(4, N = 54)_ = 16.13, p < 0.01; Mann-Whitney U: 1 d vs. 43 d, p = 0.002) (Fig 6D) whereas the median number of anti-SYNORF1-positive pixels per ROI did not change significantly with age (Kruskal-Wallis ANOVA over all groups, collar: H_(4, N = 54)_ = 8.28, p > 0.05) (Fig 6E). Interestingly, the ratio of anti-BRP^last200^-positive pixels to anti-SYNORF1-positive pixels per ROI increased significantly during the first week after emergence and from day 29 to day 43 (Kruskal-Wallis ANOVA over all groups: H_(4, N = 54)_ = 24.71, p < 0.001; Mann-Whitney U: 1 d vs. 8 d, p = 0.002; 1 d vs. 29 d, p = 0.004; 1 d vs. 43 d, p = 0.000; 15 d vs. 43 d, p = 0.002; 29 d vs. 43 d, p = 0.002) (Fig 6F).

Taken together, the median number of anti-BRP^last200^-positive pixels per ROI increased in lip and collar. In contrast, the median number of anti-SYNORF1-positive pixels per ROI decreased in the collar but did not change in the lip. In both, collar and lip, the ratio of anti-BRP^last200^-positive pixels to anti-SYNORF1-positive pixels per ROI increased with age. These findings suggest that the area, and thus presumably the amount, of the presynaptic protein AmBRP is increasing in an age-associated manner in lip and collar.

## Discussion

### AmBRP is localized near the membrane of MG boutons in the MB calyces

We demonstrate that AmBRP labeled with the BRP^last200^ antibody is detectable throughout the honeybee brain and showed a similar localization pattern as labeled Synapsin. In the MB calyces, AmBRP was found near the presynaptic cell membrane of Synapsin labeled boutons, and thus in areas where presynaptic structures are located [8, 16]. Accordingly, we conclude that BRP homologous proteins are localized at presynaptic sites of MG in the honeybee brain. Studies in *Drosophila melanogaster* and locusts reporting a presynaptic localization of BRP homologous proteins are in line with our finding [21, 22]. This suggests that AmBRP might have a similar function at bee synapses as in locusts and the fruit fly, where it is involved in the regulation of neurotransmitter release [22, 23, 37].

### Age-associated variation of AmBRP and Synapsin levels in the central brain

By using a Western blot analysis, we showed that AmBRP and Synapsin levels were significantly higher in the central brain of 15-day-old bees compared with 1-day-old bees. These high levels of AmBRP and Synapsin could be due to an age-dependent increase of the amount of these proteins in already existing presynapses or due to the formation of new, additional synapses in the central brain.

In addition to the western blot experiments, we carried out an immunohistological analysis to study the localization of AmBRP and Synapsin in the MBs. This analysis revealed predominant staining of AmBRP in the MB peduncles, which consist of bundles of axon-like processes from intrinsic MB neurons, the Kenyon cells [38]. Thus, synapses might be found along the entire peduncle. Indeed, synapses in the honeybee MB peduncle have been described already in 1971 (reviewed in Schürmann, 2016 [39]). Moreover, Rybak and Menzel (1993) [40] discovered extrinsic KC neurons, which get input from the vertical-lobe and arborize to the peduncle (as well as to the vertical-lobe, medial-lobe, and calyces of the ipsilateral MB) interconnecting subcompartments of the MBs. Based on these results and findings in other insects, Schürmann (2016) [39] suggests that the peduncle (as well as the lobes) of the MB are “sites of crosstalk” between (intrinsic) KCs and also KC extrinsic neurons.

In *Drosophila melanogaster*, Knapek et al. (2011) [41] demonstrated that DmBRP in Kenyon cells plays a critical role in the formation of an anesthesia-resistant memory: a 70% reduction of DmBRP in the Kenyon cells reduces this type of memory significantly. Accordingly, an age-associated increase of BRP, as observed in our experiment, might facilitate memory formation in fruit fly and possibly also in honeybees. However, Gupta et al. (2016) [42] demonstrated that an age-induced increase of DmBRP, which could be mimicked by an increase of the BRP copy number, did not facilitate anesthesia-resistant memory but instead blocked a cold-sensitive, anesthesia-sensitive memory. Based on these results, the authors proposed that, in the Drosophila nervous system, aging synapses might steer towards the upper limit of their operational range by increasing BRP levels. This age-dependent process might limit synaptic plasticity and contribute to impairment of memory formation with age (46).

### Age-associated variation of Synapsin and AmBRP in lip and dense collar of the MBs

Previous studies demonstrated that the packing density of boutons in lip and dense collar decreases with age resulting in fewer boutons in a defined area, i.e. a ROI, of these neuropils [8–10]. Thus, one would predict that presynaptic proteins in lip and dense collar are decreasing with age due to the decreased packing density of boutons resulting in fewer boutons per ROI that were analyzed. Indeed, this prediction proves true for Synapsin in the dense collar in our study since we observe an age-associated reduction of the number of anti-SYNORF1-positive pixels. However, this is not the case for Synapsin in the lip where the number of anti-SYNORF1-positive pixels does not change with age. What might be the reason for this finding? It was shown that, in addition to the decrease in density, the mean volume of individual boutons increases with age in the lip and the dense collar [8, 10]. This increase is stronger in the lip than in the collar [8]. Thus, the decrease in bouton density and the increase in bouton volume most likely counteract each other in the lip and this might be the reason why we see no change in the amount of Synapsin in the lip.

As it is the case with Synapsin, age-associated alterations in the structural organization of lip and collar boutons might influence the detection of anti-BRP^last200^-positive pixels. Thus, we calculated the ratio between the median number of anti-BRP^last200^-positive pixels to the median number of anti-SYNORF1-positive pixels per ROI, thereby factoring out the influence of morphological changes in the density and volume of the boutons on the detection of anti-BRP^last200^-positive pixels. The ratios, i.e. the relative area, and thus probably the amount, of AmBRP increased in an age-associated manner in both, lip and collar: In the dense collar and the lip, the relative amount of AmBRP is significantly increased in 43-day-old bees. In addition, we observe an increase in the relative amount of AmBRP in the first week after emergence in the lip.

AmBRP is a protein predominately located at presynapses (see above). Due to the age-associated increase in bouton volume (see above), boutons with a larger surface might also have more active zones. Increased numbers of active zones per bouton would lead to increased AmBRP levels which would provide an explanation for the observed age-associated increase in the relative amount of AmBRP. Indeed, this hypothesis could hold true for the collar as it was shown that the number of active zones per bouton is increased in 35-day-old bees compared with 1-day-old bees and that the proportion of ribbon vs. non-ribbon type active zones is increased in 35-day-old bees compared to 1-day-old bees. The latter is interesting, because ribbon-synapses in bees resemble T-bar-shaped synapses in fruit flies, that contain BRP, whereas non-ribbon synapses do not resemble this synapse type [8] Thus, these data are in line with our findings of an increase of AmBRP from day 1, day 8 and day 15 to day 43.

In contrast to the collar, the number of active zones per bouton remains unchanged between 1- and 35-day-old bees in the lip [8]. However, the same study showed that also in lip boutons the proportion of ribbon vs. non-ribbon type active zones increases. Thus, the AmBRP increase in the lip might not be indicative for the formation of new active zones and thus new synapses. Rather, we suggest that, in the lip, it is the amount of AmBRP at existing active zones that is altered in an age-associated manner. As mentioned above, this alteration seems to take place twice: Early after emergence and late in the bees’ lifetime between day 29 and 43. It might well be that an alteration of the amount of AmBRP at existing synapses shifts the proportion of ribbon vs non-ribbon active zones such that ribbon-active zones are increasing in an age-dependent manner.

What might be the cause of the observed age-associated alterations of AmBRP in the lip and collar? Based on the existing literature, the first increase of AmBRP in the lip could be due to maturation processes in the olfactory system. The lip can be regarded as part of this olfactory system as projection neurons from the antennal lobes convey odor information onto MB Kenyon cells in this region [12]. Neuropils belonging to the olfactory system such as the antennal lobes are not yet fully developed in newly emerged bees and mature during the first days after emergence [43–45]. These maturation processes occurring in the antennal lobe might also influence synaptic connections, and thereby probably the amount of AmBRP, in upstream odor processing centers such as the lip.

In addition to an AmBRP increase during the first week of a bee’s life, we found increased AmBRP levels in very old bees (43-day-old) in the lip, but also in the collar. Similar results were observed at neuromuscular junctions of aged fruit flies [46]. The authors found that, with progression of age, the number of BRP-labeled spots, which indicate active zones, per bouton increased up to an age of 42 days and that this increase is accompanied by an increase in bouton volume. It is known from studies on endocytosis mutants, that an increase in number of boutons and active zones compensates a decrease of synaptic vesicle exocytosis [47–49]. Thus, increased AmBRP levels at boutons in older insects might represent compensatory mechanisms for age-associated lower synaptic transmission. This hypothesis is in line with the view that age-associated synaptic alterations might be the consequence of adaptive processes due to neuronal plasticity that compensate for age-dependent cognitive impairments [50]. Indeed, work of Gupta et al. (2016) [42] demonstrated that a drop in postsynaptic excitability drives an increase of presynaptic scaffolds. According to the authors, this increase of presynaptic scaffolds might lead to an increase of synaptic vesicle release, which has been shown to be age-dependent [42]. In line, in a fruit fly model of Alzheimer’s disease, an age-dependent reduction of the amount of BRP and the synaptic vesicle release probability has been observed [51] suggesting that presynaptic β-amyloid plaques in the fruit fly brain might hinder a compensation of age-dependent processes that could be related to the amount of BRP.

### Age-associated processes and the division of labor in honeybees

A striking feature of honeybee workers is their age-related division of labor [52]. Individual workers perform different tasks within and outside the hive in an age-dependent manner: For the first 2–3 weeks after adult emergence, workers perform in-hive duties such as brood care and food processing, and start to forage for nectar and pollen outside the hive thereafter [53, 54]. This behavioral plasticity has been suggested to have both age- and experience-related determinants [55–57]. Therefore, it should be taken into account that age-associated processes observed in honeybees are not only due to their chronological age but also due to the task they fulfill because of their age and because of the state of the colony. Thus, the age-associated effects observed in this study could be due to the (unknown) age-dependent signal that triggers the switch between the two tasks, due to experiences made when fulfilling the age-associated task [58], or due to the internal state of the colony. In the latter case, the observed effects would not be due to the bees’ age but to the state of the colony. Since we studied bees of defined ages in a colony that was not manipulated, we propose that we here observe “normally” aging bees and that the effects we observe are directly or indirectly associated with the bees’ age.

Here we report that the level of the presynaptic proteins, Synapsin and AmBRP, are modified in an age-associated manner in the honeybee brain. We found an early increase in the relative amount of AmBRP during the first week after emergence in the MB lip, which we hypothesize, might be due to maturation processes in the olfactory system. We show for the first time that both MB regions, lip and collar, have increased amounts of AmBRP in 43-day-old bees. Given that BRP is homologous to the vertebrate ELKS/CAST/ERC protein [20], which is part of the presynaptic active zone, it will be interesting if these proteins are altered in an age-associated manner in vertebrates as well and if an AmBRP increase compensates for age-dependent cognitive impairments.

## Supporting information

S1 Table Data set Fig 4. (PDF)

S2 Table Data set Fig 6. (PDF)
