# Supplementary material for: Age-associated increase of the active zone protein Bruchpilot within the honeybee mushroom body
Source: PLoS One. 2017 Apr 24;12(4):e0175894. doi: 10.1371/journal.pone.0175894 (PMC5402947; doi:10.1371/journal.pone.0175894)
Supplement: S1 Table — (PDF) [file pone.0175894.s001.pdf]

S1 Table: data set Fig 4

The acquired value of the bands detected with a specific antibody (anti-BRB^{last200} (BRP), anti-SYNORF1 (Syn), anti-α-Tubulin (Tub)) was normalized to the mean value of all bands detected with this antibody on the same blot (gel-normalized). The two main AmBRP and α-Tubulin bands were measured as one band. Finally, the ratio between the normalized AmBRP and α-Tubulin values or the normalized Synapsin and α-Tubulin values was calculated for each sample (tubulin-normalized). One sample represented one bee brain.

age	bee	gel	antibody	intensity	mean intensity of antibody on gel	gel-normalized	tubulin-normalized
1	1	1	BRP	8512.70	24530.35	0.35	0.76
1	1	1	Syn	16603.00	61461.37	0.27	0.60
1	1	1	Tub	11701.67	25775.41	0.45	
8	17	1	BRP	13852.80		0.56	0.67
8	17	1	Syn	43108.36		0.70	0.84
8	17	1	Tub	21622.89		0.84	
15	28	1	BRP	20716.47		0.84	1.46
15	28	1	Syn	51183.71		0.83	1.44
15	28	1	Tub	14941.60		0.58	
29	43	1	BRP	40790.83		1.66	1.55
29	43	1	Syn	84947.25		1.38	1.29
29	43	1	Tub	27709.36		1.08	
43	58	1	BRP	19127.22		0.78	0.74
43	58	1	Syn	65293.35		1.06	1.01
43	58	1	Tub	27211.75		1.06	
1	2	1	BRP	24035.89		0.98	1.00
1	2	1	Syn	54198.49		0.88	0.90
1	2	1	Tub	25233.72		0.98	
8	18	1	BRP	23670.11		0.96	1.02
8	18	1	Syn	63317.25		1.03	1.09
8	18	1	Tub	24430.71		0.95	
15	29	1	BRP	45153.63		1.84	1.27
15	29	1	Syn	100421.12		1.63	1.12
15	29	1	Tub	37447.00		1.45	
29	44	1	BRP	26815.08		1.09	1.01
29	44	1	Syn	69540.49		1.13	1.05

29	44	1	Tub	27854.14		1.08	
43	59	1	BRP	23237.49		0.95	0.65
43	59	1	Syn	62251.44		1.01	0.69
43	59	1	Tub	37642.71		1.46	
1	3	1	BRP	37686.80		1.54	1.16
1	3	1	Syn	89315.11		1.45	1.10
1	3	1	Tub	34006.67		1.32	
8	19	1	BRP	10765.16		0.44	0.58
8	19	1	Syn	37356.86		0.61	0.80
8	19	1	Tub	19502.72		0.76	
15	30	2	BRP	10390.69	25054.30	0.41	0.41
15	30	2	Syn	88555.54	76186.47	1.16	1.16
15	30	2	Tub	32596.41	32507.35	1.00	
29	45	2	BRP	8583.53		0.34	0.49
29	45	2	Syn	59812.20		0.79	1.11
29	45	2	Tub	22905.23		0.70	
43	60	2	BRP	21527.45		0.86	0.83
43	60	2	Tub	33613.53		1.03	
1	4	2	BRP	22484.19		0.90	0.94
1	4	2	Tub	30996.33		0.95	
8	20	2	BRP	9641.22		0.38	0.59
8	20	2	Tub	21041.65		0.65	
15	31	2	BRP	37358.65		1.49	1.29
15	31	2	Tub	37695.82		1.16	
29	46	2	BRP	42760.69		1.71	1.03
29	46	2	Tub	54064.76		1.66	
43	61	2	BRP	59853.64		2.39	1.71
43	61	2	Tub	45350.42		1.40	
1	5	2	BRP	25334.08		1.01	1.12
1	5	2	Tub	29376.14		0.90	
8	21	2	BRP	14768.63		0.59	0.98
8	21	2	Tub	19484.79		0.60	
15	32	2	BRP	35734.09		1.43	1.68
15	32	2	Syn	90002.26		1.18	1.39
15	32	2	Tub	27673.56		0.85	
29	47	2	BRP	27174.58		1.08	0.94
29	47	2	Syn	88867.67		1.17	1.01

29	47	2	Tub	37357.91		1.15	
43	62	2	BRP	10094.50		0.40	0.43
43	62	2	Syn	53694.67		0.70	0.75
43	62	2	Tub	30439.00		0.94	
1	6	3	BRP	12503.79	34347.38	0.36	0.36
1	6	3	Syn	37769.09	63100.18	0.60	0.60
1	6	3	Tub	25173.56	25105.60	1.00	
8	22	3	BRP	12730.87		0.37	0.63
8	22	3	Syn	36869.04		0.58	1.00
8	22	3	Tub	14681.65		0.58	
15	33	3	BRP	31762.50		0.92	0.98
15	33	3	Syn	85077.00		1.35	1.43
15	33	3	Tub	23685.00		0.94	
29	48	3	BRP	49015.31		1.43	0.97
29	48	3	Syn	100209.58		1.59	1.08
29	48	3	Tub	36973.60		1.47	
43	63	3	BRP	50528.19		1.47	1.26
43	63	3	Syn	90273.20		1.43	1.23
43	63	3	Tub	29315.59		1.17	
1	7	3	BRP	26741.98		0.78	0.76
1	7	3	Syn	47105.00		0.75	0.73
1	7	3	Tub	25787.23		1.03	
8	23	3	BRP	37970.09		1.11	1.09
8	23	3	Syn	52874.57		0.84	0.83
8	23	3	Tub	25383.09		1.01	
15	34	3	BRP	31901.38		0.93	0.93
15	34	3	Syn	49162.27		0.78	0.78
15	34	3	Tub	24947.95		0.99	
29	49	3	BRP	22887.33		0.67	0.57
29	49	3	Syn	69950.36		1.11	0.96
29	49	3	Tub	29096.50		1.16	
43	64	3	BRP	70580.35		2.05	1.85
43	64	3	Syn	98177.82		1.56	1.40
43	64	3	Tub	27957.00		1.11	
1	8	3	BRP	23082.29		0.67	0.87
1	8	3	Syn	42365.61		0.67	0.87
1	8	3	Tub	19390.92		0.77	

8	24	3	BRP	42464.50		1.24	1.64
8	24	3	Syn	47368.64		0.75	1.00
8	24	3	Tub	18875.12		0.75	
15	35	4	BRP	27173.95	32460.28	0.79	0.78
15	35	4	Syn	85242.74	64126.98	1.23	1.21
15	35	4	Tub	32538.82	29958.07	1.02	
29	50	4	BRP	19172.15		0.56	1.08
29	50	4	Syn	57231.17		0.83	1.59
29	50	4	Tub	16656.03		0.52	
43	65	4	BRP	30834.84		0.90	0.69
43	65	4	Syn	95192.47		1.38	1.06
43	65	4	Tub	41572.40		1.30	
1	9	4	BRP	18247.00		0.53	0.61
1	9	4	Syn	48659.75		0.70	0.81
1	9	4	Tub	27770.32		0.87	
15	36	4	BRP	39763.26		1.16	1.31
15	36	4	Syn	84958.33		1.23	1.39
15	36	4	Tub	28379.51		0.89	
29	51	4	BRP	43307.95		1.27	0.95
29	51	4	Syn	68402.30		0.99	0.75
29	51	4	Tub	42476.70		1.33	
43	66	4	BRP	80973.46		2.37	1.29
43	66	4	Syn	129423.31		1.87	1.02
43	66	4	Tub	58564.08		1.83	
1	10	4	BRP	10276.31		0.30	0.43
1	10	4	Syn	22561.69		0.33	0.47
1	10	4	Tub	22122.82		0.69	
8	26	4	BRP	28303.59		0.83	0.90
8	26	4	Syn	49950.66		0.72	0.79
8	26	4	Tub	29373.62		0.92	
15	37	4	BRP	61174.10		1.79	1.58
15	37	4	Syn	82882.52		1.20	1.06
15	37	4	Tub	36169.22		1.13	
29	52	4	BRP	17098.00		0.50	0.99
29	52	4	Syn	35527.16		0.51	1.02
29	52	4	Tub	16128.29		0.50	
1	11	5	BRP	7685.00	18688.75	0.41	0.57

1	11	5	Syn	23506.75	69342.05	0.34	0.47
1	11	5	Tub	18597.32	25830.81	0.72	
8	38	5	BRP	5906.50		0.32	0.77
8	38	5	Syn	21828.09		0.31	0.77
8	38	5	Tub	10546.50		0.41	
15	39	5	BRP	22096.81		1.18	1.68
15	39	5	Syn	77900.31		1.12	1.60
15	39	5	Tub	18160.29		0.70	
29	53	5	BRP	34228.33		1.83	1.71
29	53	5	Syn	107470.13		1.55	1.45
29	53	5	Tub	27671.43		1.07	
43	67	5	BRP	22604.27		1.21	1.11
43	67	5	Syn	95578.17		1.38	1.26
43	67	5	Tub	28238.44		1.09	
1	12	5	BRP	6294.40		0.34	0.46
1	12	5	Syn	33300.13		0.48	0.66
1	12	5	Tub	18770.00		0.73	
8	27	5	BRP	5958.54		0.32	0.51
8	27	5	Syn	29926.29		0.43	0.69
8	27	5	Tub	16225.79		0.63	
15	40	5	BRP	24453.43		1.31	0.76
15	40	5	Syn	93290.32		1.35	0.79
15	40	5	Tub	44245.00		1.71	
29	54	5	BRP	22774.37		1.22	0.75
29	54	5	Syn	90430.69		1.30	0.80
29	54	5	Tub	42165.63		1.63	
43	68	5	BRP	37226.51		1.99	1.24
43	68	5	Syn	133570.63		1.93	1.20
43	68	5	Tub	41343.75		1.60	
1	13	5	BRP	5550.46		0.30	0.78
1	13	5	Syn	14080.01		0.20	0.54
1	13	5	Tub	9778.12		0.38	
15	41	5	BRP	29486.40		1.58	1.19
15	41	5	Syn	111223.04		1.60	1.21
15	41	5	Tub	34227.50		1.33	
29	55	6	BRP	4520.73	16806.79	0.27	0.48
29	55	6	Syn	34327.40	69745.10	0.49	0.89

29	55	6	Tub	16092.54	29005.67	0.55	
43	69	6	BRP	10418.41		0.62	0.78
43	69	6	Syn	65653.33		0.94	1.18
43	69	6	Tub	23182.58		0.80	
1	14	6	BRP	7282.50		0.43	0.50
1	14	6	Syn	43155.60		0.62	0.71
1	14	6	Tub	25240.47		0.87	
15	42	6	BRP	34547.48		2.06	1.71
15	42	6	Tub	34855.97		1.20	
29	56	6	BRP	14391.11		0.86	1.52
29	56	6	Tub	16378.17		0.56	
43	70	6	BRP	16568.00		0.99	0.77
43	70	6	Syn	67561.00		0.97	0.75
43	70	6	Tub	37301.50		1.29	
1	15	6	BRP	7978.89		0.47	0.46
1	15	6	Syn	45359.12		0.65	0.63
1	15	6	Tub	29830.70		1.03	
29	57	6	BRP	46603.16		2.77	1.72
29	57	6	Syn	155874.44		2.23	1.38
29	57	6	Tub	46857.50		1.62	
43	71	6	BRP	11963.93		0.71	0.71
43	71	6	Syn	64379.08		0.92	0.93
43	71	6	Tub	28934.75		1.00	
1	16	6	BRP	15015.19		0.89	0.79
1	16	6	Syn	57623.42		0.83	0.73
1	16	6	Tub	32815.42		1.13	
43	72	6	BRP	26646.03		1.59	1.74
43	72	6	Syn	92019.92		1.32	1.45
43	72	6	Tub	26416.79		0.91	
43	73	6	BRP	5746.00		0.34	0.33
43	73	6	Syn	71497.69		1.03	0.99
43	73	6	Tub	30161.63		1.04	
