# Supplementary material for: Age-associated increase of the active zone protein Bruchpilot within the honeybee mushroom body
Source: PLoS One. 2017 Apr 24;12(4):e0175894. doi: 10.1371/journal.pone.0175894 (PMC5402947; doi:10.1371/journal.pone.0175894)
Supplement: S2 Table — (PDF) [file pone.0175894.s002.pdf]

S2 Table: data set Fig 6

In order to measure those areas within the MG boutons in lip and collar containing anti-BRPlast200 and anti-SYNORF1 signals, we placed two regions of interest (ROIs) in the dense collar (because of the uneven distribution of boutons in the loose-collar region) and two ROIs in the lip region of both medial calyces in the scanned images. Each ROI covers a square area of 400 µm² (86 x 86 pixels, pixel size 232 nm). The signal intensity of each pixel in aper ROI was measured separately for the anti-BRPlast200 and the anti-SYNORF1 staining, i.e. for each channel. Next, the lowest intensity value of a channel measured in aper ROI was subtracted from all other values in this ROI. Each of these background corrected values was normalized to the highest calculated value in its ROI to obtain relative values between zero and one. To detect those pixels indicating the presence of an antibody staining, each relative value that was higher than an antibody- and region-specific threshold was defined as signal (anti-BRPlast200-positive or anti-SYNORF1-positive pixels). This threshold was defined as the sum of a constant, antibody- or dye-specific value (0.1 for Alexa Fluor 546 Phalloidin, 0.05 for anti-SYNORF1, and 0.15 for anti-BRPlast200, constant values were set by the experimenter) and the median of all values in one ROI. In doing so, potential discrepancies in value intensities between different ROIs based on the staining method and between sample differences were taken into account. For figure 6, we calculated the median number of anti-BRPlast200- and anti-SYNORF1-positive pixels per ROI and the ratio between the two medians for collar and lip. The calculated number of Anti-SYNORF1 and Anti-BRP positive pixels for each measured ROI in each bee, the mean number of Anti-SYNORF1 and Anti-BRPlast200 positive pixels lip and dense collar in each bee and their ratio is shown in this table. *MB: medical calyx (mc) number according to figure 05 (1=mc1, 2=mc2, 3=mc3, 4=mc4),** ROI: nomenclature of ROIs measured (lip ROI1=l1, lip ROI 2=l2, dense collar ROI 3=c1, dense collar ROI 4=c2)

age	bee	MB*	ROI**	number Anti-SYNORF1 pixel per ROI	number Anti-BRP pixel per ROI		MB region	mean number Anti-SYNORF1 pixel per bee and region	mean number Anti-BRP per pixel per bee and region	Anti-BRP/Anti-SYNORF1 pixel per ROI per bee and region
01d	1	mc1	c1	2369	443		collar	2575.25	547.38	0.21
01d	1	mc2	c1	2701	327		lip	2222.38	778.38	0.35
01d	1	mc3	c1	2567	343					
01d	1	mc4	c1	2552	625					
01d	1	mc1	c2	2461	818					
01d	1	mc2	c2	2780	572					
01d	1	mc3	c2	2513	834					
01d	1	mc4	c2	2659	417					
01d	1	mc1	l1	2108	879					
01d	1	mc2	l1	2251	917					
01d	1	mc3	l1	2260	785					
01d	1	mc4	l1	1954	625					
01d	1	mc1	l2	2532	894					
01d	1	mc2	l2	2291	826					

01d	1	mc3	l2	2054	583					
01d	1	mc4	l2	2329	718					
01d	2	mc1	c1	2110	573		collar	2310.75	588.88	0.25
01d	2	mc2	c1	2490	811		lip	2122.88	656.63	0.31
01d	2	mc3	c1	2526	512					
01d	2	mc4	c1	2254	452					
01d	2	mc1	c2	2289	705					
01d	2	mc2	c2	2402	653					
01d	2	mc3	c2	2136	341					
01d	2	mc4	c2	2279	664					
01d	2	mc1	l1	2338	817					
01d	2	mc2	l1	2357	596					
01d	2	mc3	l1	2331	401					
01d	2	mc4	l1	1781	534					
01d	2	mc1	l2	1584	826					
01d	2	mc2	l2	2036	942					
01d	2	mc3	l2	2221	442					
01d	2	mc4	l2	2335	695					
01d	3	mc1	c1	2221	865		collar	2369.25	699.25	0.30
01d	3	mc2	c1	2539	597		lip	2177.75	651.00	0.30
01d	3	mc3	c1	2376	450					
01d	3	mc4	c1	2308	726					
01d	3	mc1	c2	2298	730					
01d	3	mc2	c2	2336	652					
01d	3	mc3	c2	2610	724					
01d	3	mc4	c2	2266	850					
01d	3	mc1	l1	1612	439					
01d	3	mc2	l1	2615	377					
01d	3	mc3	l1	2521	771					
01d	3	mc4	l1	2028	811					
01d	3	mc1	l2	2385	587					
01d	3	mc2	l2	1851	416					
01d	3	mc3	l2	2299	907					
01d	3	mc4	l2	2111	900					
01d	4	mc1	c1	2285	614		collar	2476.38	525.38	0.21
01d	4	mc2	c1	2569	623		lip	1941.25	560.25	0.29
01d	4	mc3	c1	2468	688					

01d	4	mc4	c1	2418	444					
01d	4	mc1	c2	2411	592					
01d	4	mc2	c2	2618	363					
01d	4	mc3	c2	2621	447					
01d	4	mc4	c2	2421	432					
01d	4	mc1	l1	1530	390					
01d	4	mc2	l1	2147	514					
01d	4	mc3	l1	2076	412					
01d	4	mc4	l1	2061	362					
01d	4	mc1	l2	1637	786					
01d	4	mc2	l2	2131	548					
01d	4	mc3	l2	1986	675					
01d	4	mc4	l2	1962	795					
01d	5	mc1	c1	2423	459		collar	2520.75	459.63	0.18
01d	5	mc2	c1	2340	810		lip	2044.88	399.38	0.20
01d	5	mc3	c1	2498	837					
01d	5	mc4	c1	2657	332					
01d	5	mc1	c2	2404	191					
01d	5	mc2	c2	2440	249					
01d	5	mc3	c2	2528	371					
01d	5	mc4	c2	2876	428					
01d	5	mc1	l1	1915	460					
01d	5	mc2	l1	2138	274					
01d	5	mc3	l1	2177	261					
01d	5	mc4	l1	2123	358					
01d	5	mc1	l2	1903	425					
01d	5	mc2	l2	1961	378					
01d	5	mc3	l2	2072	534					
01d	5	mc4	l2	2070	505					
01d	6	mc1	c1	2279	528		collar	2342.50	596.38	0.25
01d	6	mc2	c1	2379	458		lip	1989.38	675.75	0.34
01d	6	mc3	c1	2289	621					
01d	6	mc4	c1	2415	414					
01d	6	mc1	c2	2488	910					
01d	6	mc2	c2	2327	749					
01d	6	mc3	c2	2214	522					
01d	6	mc4	c2	2349	569					

01d	6	mc1	l1	2072	568					
01d	6	mc2	l1	1947	738					
01d	6	mc3	l1	1897	518					
01d	6	mc4	l1	1888	723					
01d	6	mc1	l2	2054	881					
01d	6	mc2	l2	2219	910					
01d	6	mc3	l2	1779	533					
01d	6	mc4	l2	2059	535					
01d	7	mc1	c1	2537	588		collar	2558.38	581.13	0.23
01d	7	mc2	c1	2678	530		lip	1980.50	622.00	0.31
01d	7	mc3	c1	2414	709					
01d	7	mc4	c1	2469	487					
01d	7	mc1	c2	2523	559					
01d	7	mc2	c2	2846	586					
01d	7	mc3	c2	2530	562					
01d	7	mc4	c2	2470	628					
01d	7	mc1	l1	1839	624					
01d	7	mc2	l1	2292	578					
01d	7	mc3	l1	2161	480					
01d	7	mc4	l1	1819	599					
01d	7	mc1	l2	1945	845					
01d	7	mc2	l2	2205	845					
01d	7	mc3	l2	1724	387					
01d	7	mc4	l2	1859	618					
01d	8	mc1	c1	2180	626		collar	2368.75	549.13	0.23
01d	8	mc2	c1	2295	647		lip	1972.38	460.50	0.23
01d	8	mc3	c1	2368	774					
01d	8	mc4	c1	2141	581					
01d	8	mc1	c2	2512	814					
01d	8	mc2	c2	2499	327					
01d	8	mc3	c2	2478	346					
01d	8	mc4	c2	2477	278					
01d	8	mc1	l1	1598	736					
01d	8	mc2	l1	2030	471					
01d	8	mc3	l1	2144	519					
01d	8	mc4	l1	2058	336					
01d	8	mc1	l2	1562	450					

01d	8	mc2	l2	2122	330					
01d	8	mc3	l2	2144	343					
01d	8	mc4	l2	2121	499					
01d	9	mc1	c1	2277	935		collar	2366.38	706.25	0.30
01d	9	mc2	c1	2282	596		lip	1809.00	759.88	0.42
01d	9	mc3	c1	2351	585					
01d	9	mc4	c1	2453	592					
01d	9	mc1	c2	2415	578					
01d	9	mc2	c2	2339	735					
01d	9	mc3	c2	2287	674					
01d	9	mc4	c2	2527	955					
01d	9	mc1	l1	1784	886					
01d	9	mc2	l1	1762	732					
01d	9	mc3	l1	1926	741					
01d	9	mc4	l1	1929	559					
01d	9	mc1	l2	1456	718					
01d	9	mc2	l2	1853	825					
01d	9	mc3	l2	1877	837					
01d	9	mc4	l2	1885	781					
08d	2	mc1	c1	2309	518		collar	2296.38	482.00	0.21
08d	2	mc2	c1	2549	610		lip	1793.25	696.13	0.39
08d	2	mc3	c1	2006	669					
08d	2	mc4	c1	2259	411					
08d	2	mc1	c2	2364	583					
08d	2	mc2	c2	2352	250					
08d	2	mc3	c2	2131	584					
08d	2	mc4	c2	2401	231					
08d	2	mc1	l1	1396	406					
08d	2	mc2	l1	1869	676					
08d	2	mc3	l1	2124	996					
08d	2	mc4	l1	2124	605					
08d	2	mc1	l2	1583	546					
08d	2	mc2	l2	1571	749					
08d	2	mc3	l2	1746	799					
08d	2	mc4	l2	1933	792					
08d	3	mc1	c1	2335	918		collar	2248.25	649.38	0.29
08d	3	mc2	c1	2213	605		lip	1858.38	675.00	0.36

08d	3	mc3	c1	2050	583					
08d	3	mc4	c1	2373	625					
08d	3	mc1	c2	2285	865					
08d	3	mc2	c2	2258	453					
08d	3	mc3	c2	1999	681					
08d	3	mc4	c2	2473	465					
08d	3	mc1	l1	2173	502					
08d	3	mc2	l1	1794	593					
08d	3	mc3	l1	2050	915					
08d	3	mc4	l1	1776	600					
08d	3	mc1	l2	1803	583					
08d	3	mc2	l2	1497	567					
08d	3	mc3	l2	1999	854					
08d	3	mc4	l2	1775	786					
08d	4	mc1	c1	2046	1031		collar	2149.13	610.88	0.28
08d	4	mc2	c1	1933	389		lip	1651.00	746.25	0.45
08d	4	mc3	c1	2255	663					
08d	4	mc4	c1	2332	452					
08d	4	mc1	c2	2207	429					
08d	4	mc2	c2	2107	907					
08d	4	mc3	c2	1921	535					
08d	4	mc4	c2	2392	481					
08d	4	mc1	l1	2239	713					
08d	4	mc2	l1	1446	546					
08d	4	mc3	l1	1489	727					
08d	4	mc4	l1	1413	490					
08d	4	mc1	l2	1816	737					
08d	4	mc2	l2	1728	779					
08d	4	mc3	l2	1540	1081					
08d	4	mc4	l2	1537	897					
08d	5	mc1	c1	1965	840		collar	2157.00	667.88	0.31
08d	5	mc2	c1	2144	1079		lip	1512.38	670.75	0.44
08d	5	mc3	c1	2261	398					
08d	5	mc4	c1	2111	265					
08d	5	mc1	c2	2345	648					
08d	5	mc2	c2	2103	1042					
08d	5	mc3	c2	2025	592					

08d	5	mc4	c2	2302	479					
08d	5	mc1	l1	1468	564					
08d	5	mc2	l1	1427	517					
08d	5	mc3	l1	1724	959					
08d	5	mc4	l1	1654	524					
08d	5	mc1	l2	1134	526					
08d	5	mc2	l2	1567	996					
08d	5	mc3	l2	1677	756					
08d	5	mc4	l2	1448	524					
08d	6	mc1	c1	2273	643		collar	2429.63	585.50	0.24
08d	6	mc2	c1	2251	422		lip	1849.88	621.13	0.34
08d	6	mc3	c1	2423	749					
08d	6	mc4	c1	2690	472					
08d	6	mc1	c2	2530	589					
08d	6	mc2	c2	2292	681					
08d	6	mc3	c2	2259	551					
08d	6	mc4	c2	2719	577					
08d	6	mc1	l1	1965	675					
08d	6	mc2	l1	2037	528					
08d	6	mc3	l1	1653	493					
08d	6	mc4	l1	1843	683					
08d	6	mc1	l2	1970	750					
08d	6	mc2	l2	1724	609					
08d	6	mc3	l2	1639	626					
08d	6	mc4	l2	1968	605					
08d	7	mc1	c1	2498	610		collar	2484.75	542.88	0.22
08d	7	mc2	c1	2388	547		lip	1739.75	834.63	0.48
08d	7	mc3	c1	2300	335					
08d	7	mc4	c1	2507	443					
08d	7	mc1	c2	2563	790					
08d	7	mc2	c2	2606	416					
08d	7	mc3	c2	2427	384					
08d	7	mc4	c2	2589	818					
08d	7	mc1	l1	1912	787					
08d	7	mc2	l1	1860	851					
08d	7	mc3	l1	1433	946					
08d	7	mc4	l1	1685	666					

08d	7	mc1	l2	2056	976					
08d	7	mc2	l2	1881	741					
08d	7	mc3	l2	1589	903					
08d	7	mc4	l2	1502	807					
08d	8	mc1	c1	2299	985		collar	2390.25	734.00	0.31
08d	8	mc2	c1	2447	732		lip	1957.63	812.88	0.42
08d	8	mc3	c1	2365	494					
08d	8	mc4	c1	2473	591					
08d	8	mc1	c2	2394	706					
08d	8	mc2	c2	2270	690					
08d	8	mc3	c2	2374	1079					
08d	8	mc4	c2	2500	595					
08d	8	mc1	l1	1862	804					
08d	8	mc2	l1	1906	805					
08d	8	mc3	l1	1772	645					
08d	8	mc4	l1	2109	872					
08d	8	mc1	l2	1750	1166					
08d	8	mc2	l2	2237	878					
08d	8	mc3	l2	1994	506					
08d	8	mc4	l2	2031	827					
08d	9	mc1	c1	2493	945		collar	2358.88	759.38	0.32
08d	9	mc2	c1	2483	842		lip	1979.88	913.63	0.46
08d	9	mc3	c1	2435	756					
08d	9	mc4	c1	2460	664					
08d	9	mc1	c2	2281	895					
08d	9	mc2	c2	2146	474					
08d	9	mc3	c2	2193	693					
08d	9	mc4	c2	2380	806					
08d	9	mc1	l1	1267	499					
08d	9	mc2	l1	2333	946					
08d	9	mc3	l1	2480	946					
08d	9	mc4	l1	1402	1005					
08d	9	mc1	l2	1904	1057					
08d	9	mc2	l2	2344	1081					
08d	9	mc3	l2	1751	920					
08d	9	mc4	l2	2358	855					
08d	10	mc1	c1	2256	648		collar	2293.25	676.50	0.29

08d	10	mc2	c1	2254	634		lip	1996.50	819.13	0.41
08d	10	mc3	c1	2226	791					
08d	10	mc4	c1	2283	736					
08d	10	mc1	c2	2292	755					
08d	10	mc2	c2	2379	740					
08d	10	mc3	c2	2380	498					
08d	10	mc4	c2	2276	610					
08d	10	mc1	l1	1559	554					
08d	10	mc2	l1	2201	932					
08d	10	mc3	l1	1640	894					
08d	10	mc4	l1	2203	725					
08d	10	mc1	l2	1925	424					
08d	10	mc2	l2	2055	1019					
08d	10	mc3	l2	2228	966					
08d	10	mc4	l2	2161	1039					
08d	11	mc1	c1	2412	695		collar	2447.88	622.25	0.25
08d	11	mc2	c1	2533	340		lip	1948.00	702.63	0.36
08d	11	mc3	c1	2271	925					
08d	11	mc4	c1	2581	477					
08d	11	mc1	c2	2362	375					
08d	11	mc2	c2	2471	584					
08d	11	mc3	c2	2417	905					
08d	11	mc4	c2	2536	677					
08d	11	mc1	l1	1758	760					
08d	11	mc2	l1	1913	718					
08d	11	mc3	l1	1850	660					
08d	11	mc4	l1	1972	528					
08d	11	mc1	l2	1805	910					
08d	11	mc2	l2	2136	713					
08d	11	mc3	l2	2177	584					
08d	11	mc4	l2	1973	748					
08d	12	mc1	c1	2419	1302		collar	2487.13	874.00	0.35
08d	12	mc2	c1	2387	896		lip	2081.38	942.75	0.45
08d	12	mc3	c1	2395	631					
08d	12	mc4	c1	2606	1075					
08d	12	mc1	c2	2534	941					
08d	12	mc2	c2	2554	751					

08d	12	mc3	c2	2464	529					
08d	12	mc4	c2	2538	867					
08d	12	mc1	l1	1619	1030					
08d	12	mc2	l1	2427	710					
08d	12	mc3	l1	2094	826					
08d	12	mc4	l1	1945	750					
08d	12	mc1	l2	2377	984					
08d	12	mc2	l2	2277	1148					
08d	12	mc3	l2	1835	1107					
08d	12	mc4	l2	2077	987					
15d	1	mc1	c1	2277	644		collar	2586.88	626.25	0.24
15d	1	mc2	c1	2632	728		lip	2075.00	612.50	0.30
15d	1	mc3	c1	2537	329					
15d	1	mc4	c1	2722	578					
15d	1	mc1	c2	2584	899					
15d	1	mc2	c2	2767	268					
15d	1	mc3	c2	2551	825					
15d	1	mc4	c2	2625	739					
15d	1	mc1	l1	1960	824					
15d	1	mc2	l1	2098	593					
15d	1	mc3	l1	2304	532					
15d	1	mc4	l1	1636	489					
15d	1	mc1	l2	2217	726					
15d	1	mc2	l2	2045	487					
15d	1	mc3	l2	2150	701					
15d	1	mc4	l2	2190	548					
15d	2	mc1	c1	2551	749		collar	2495.75	827.75	0.33
15d	2	mc2	c1	2589	1011		lip	2086.75	890.50	0.43
15d	2	mc3	c1	2558	956					
15d	2	mc4	c1	2431	863					
15d	2	mc1	c2	2650	842					
15d	2	mc2	c2	2453	882					
15d	2	mc3	c2	2397	569					
15d	2	mc4	c2	2337	750					
15d	2	mc1	l1	2143	993					
15d	2	mc2	l1	2156	1181					
15d	2	mc3	l1	2076	558					

15d	2	mc4	l1	1565	911					
15d	2	mc1	l2	2253	820					
15d	2	mc2	l2	2374	962					
15d	2	mc3	l2	1902	659					
15d	2	mc4	l2	2225	1040					
15d	3	mc1	c1	2722	198		collar	2546.38	581.38	0.23
15d	3	mc2	c1	2523	448		lip	1932.25	642.38	0.33
15d	3	mc3	c1	2693	645					
15d	3	mc4	c1	2335	692					
15d	3	mc1	c2	2658	807					
15d	3	mc2	c2	2503	278					
15d	3	mc3	c2	2457	793					
15d	3	mc4	c2	2480	790					
15d	3	mc1	l1	1844	565					
15d	3	mc2	l1	1914	673					
15d	3	mc3	l1	1752	662					
15d	3	mc4	l1	1802	770					
15d	3	mc1	l2	2152	596					
15d	3	mc2	l2	1973	884					
15d	3	mc3	l2	2428	514					
15d	3	mc4	l2	1593	475					
15d	4	mc1	c1	2774	389		collar	2607.38	597.88	0.23
15d	4	mc2	c1	2464	761		lip	1881.88	765.63	0.41
15d	4	mc3	c1	2470	821					
15d	4	mc4	c1	2687	369					
15d	4	mc1	c2	2604	161					
15d	4	mc2	c2	2701	662					
15d	4	mc3	c2	2441	1099					
15d	4	mc4	c2	2718	521					
15d	4	mc1	l1	1686	1069					
15d	4	mc2	l1	1846	618					
15d	4	mc3	l1	2259	839					
15d	4	mc4	l1	1902	840					
15d	4	mc1	l2	1965	640					
15d	4	mc2	l2	1766	700					
15d	4	mc3	l2	1831	635					
15d	4	mc4	l2	1800	784					

15d	5	mc1	c1	2258	880		collar	2315.88	806.63	0.35
15d	5	mc2	c1	2148	825		lip	2071.88	919.88	0.44
15d	5	mc3	c1	2334	917					
15d	5	mc4	c1	2459	587					
15d	5	mc1	c2	2240	896					
15d	5	mc2	c2	2389	682					
15d	5	mc3	c2	2111	826					
15d	5	mc4	c2	2588	840					
15d	5	mc1	l1	1858	938					
15d	5	mc2	l1	1984	937					
15d	5	mc3	l1	2296	914					
15d	5	mc4	l1	1922	848					
15d	5	mc1	l2	2159	895					
15d	5	mc2	l2	2016	1093					
15d	5	mc3	l2	2291	1015					
15d	5	mc4	l2	2049	719					
15d	6	mc1	c1	1985	417		collar	2404.00	555.25	0.23
15d	6	mc2	c1	2528	621		lip	2044.00	754.75	0.37
15d	6	mc3	c1	2394	665					
15d	6	mc4	c1	2637	595					
15d	6	mc1	c2	2174	522					
15d	6	mc2	c2	2397	570					
15d	6	mc3	c2	2538	690					
15d	6	mc4	c2	2579	362					
15d	6	mc1	l1	2527	449					
15d	6	mc2	l1	1845	560					
15d	6	mc3	l1	1682	576					
15d	6	mc4	l1	2071	920					
15d	6	mc1	l2	1741	875					
15d	6	mc2	l2	2137	872					
15d	6	mc3	l2	2273	1021					
15d	6	mc4	l2	2076	765					
15d	7	mc1	c1	2510	784		collar	2511.88	689.75	0.27
15d	7	mc2	c1	2642	970		lip	1886.00	749.88	0.40
15d	7	mc3	c1	2399	746					
15d	7	mc4	c1	2569	716					
15d	7	mc1	c2	2420	470					

15d	7	mc2	c2	2470	295					
15d	7	mc3	c2	2431	798					
15d	7	mc4	c2	2654	739					
15d	7	mc1	l1	1622	623					
15d	7	mc2	l1	1735	841					
15d	7	mc3	l1	2179	938					
15d	7	mc4	l1	1822	587					
15d	7	mc1	l2	2127	662					
15d	7	mc2	l2	1781	860					
15d	7	mc3	l2	2089	889					
15d	7	mc4	l2	1733	599					
15d	9	mc1	c1	2396	400		collar	2432.25	496.88	0.20
15d	9	mc2	c1	2421	92		lip	1802.63	684.13	0.38
15d	9	mc3	c1	2450	474					
15d	9	mc4	c1	2413	356					
15d	9	mc1	c2	2346	481					
15d	9	mc2	c2	2441	927					
15d	9	mc3	c2	2489	564					
15d	9	mc4	c2	2502	681					
15d	9	mc1	l1	1591	395					
15d	9	mc2	l1	1815	870					
15d	9	mc3	l1	1637	415					
15d	9	mc4	l1	1313	491					
15d	9	mc1	l2	1534	767					
15d	9	mc2	l2	2330	926					
15d	9	mc3	l2	1959	758					
15d	9	mc4	l2	2242	851					
15d	10	mc1	c1	2676	555		collar	2670.13	640.13	0.24
15d	10	mc2	c1	2599	769		lip	2020.50	762.88	0.38
15d	10	mc3	c1	2605	632					
15d	10	mc4	c1	2754	254					
15d	10	mc1	c2	2715	484					
15d	10	mc2	c2	2696	888					
15d	10	mc3	c2	2646	802					
15d	10	mc4	c2	2670	737					
15d	10	mc1	l1	1770	657					
15d	10	mc2	l1	1993	766					

15d	10	mc3	l1	2204	589					
15d	10	mc4	l1	2051	802					
15d	10	mc1	l2	1956	893					
15d	10	mc2	l2	2233	595					
15d	10	mc3	l2	1995	1066					
15d	10	mc4	l2	1962	735					
15d	11	mc1	c1	1755	590		collar	2312.25	627.38	0.27
15d	11	mc2	c1	2358	818		lip	2005.63	704.00	0.35
15d	11	mc3	c1	2477	725					
15d	11	mc4	c1	2583	265					
15d	11	mc1	c2	1959	750					
15d	11	mc2	c2	2460	469					
15d	11	mc3	c2	2340	928					
15d	11	mc4	c2	2566	474					
15d	11	mc1	l1	1742	833					
15d	11	mc2	l1	1734	807					
15d	11	mc3	l1	2310	650					
15d	11	mc4	l1	2096	866					
15d	11	mc1	l2	1744	502					
15d	11	mc2	l2	1885	611					
15d	11	mc3	l2	2173	561					
15d	11	mc4	l2	2361	802					
29d	1	mc1	c1	2310	253		collar	2432.38	493.75	0.20
29d	1	mc2	c1	2469	164		lip	1918.75	740.38	0.39
29d	1	mc3	c1	2516	637					
29d	1	mc4	c1	2431	759					
29d	1	mc1	c2	2333	577					
29d	1	mc2	c2	2252	797					
29d	1	mc3	c2	2487	477					
29d	1	mc4	c2	2661	286					
29d	1	mc1	l1	1928	708					
29d	1	mc2	l1	1841	640					
29d	1	mc3	l1	2094	559					
29d	1	mc4	l1	1946	858					
29d	1	mc1	l2	2086	713					
29d	1	mc2	l2	1766	363					
29d	1	mc3	l2	1867	997					

29d	1	mc4	l2	1822	1085					
29d	2	mc1	c1	1868	904		collar	2298.50	903.38	0.39
29d	2	mc2	c1	2417	1015		lip	2050.38	832.25	0.41
29d	2	mc3	c1	2193	939					
29d	2	mc4	c1	2310	714					
29d	2	mc1	c2	2408	766					
29d	2	mc2	c2	2500	827					
29d	2	mc3	c2	2364	1115					
29d	2	mc4	c2	2328	947					
29d	2	mc1	l1	1596	707					
29d	2	mc2	l1	2005	797					
29d	2	mc3	l1	1958	928					
29d	2	mc4	l1	2198	734					
29d	2	mc1	l2	2238	640					
29d	2	mc2	l2	2383	748					
29d	2	mc3	l2	2015	1067					
29d	2	mc4	l2	2010	1037					
29d	3	mc1	c1	1439	229		collar	2364.63	493.88	0.21
29d	3	mc2	c1	2689	673		lip	1933.63	645.00	0.33
29d	3	mc3	c1	2682	747					
29d	3	mc4	c1	2594	762					
29d	3	mc1	c2	1501	272					
29d	3	mc2	c2	2695	559					
29d	3	mc3	c2	2609	445					
29d	3	mc4	c2	2708	264					
29d	3	mc1	l1	2573	252					
29d	3	mc2	l1	1785	371					
29d	3	mc3	l1	1976	654					
29d	3	mc4	l1	1477	564					
29d	3	mc1	l2	2439	946					
29d	3	mc2	l2	1759	508					
29d	3	mc3	l2	1756	982					
29d	3	mc4	l2	1704	883					
29d	4	mc1	c1	2395	686		collar	2265.88	725.75	0.32
29d	4	mc2	c1	2253	655		lip	2207.38	894.63	0.41
29d	4	mc3	c1	2294	920					
29d	4	mc4	c1	2166	572					

29d	4	mc1	c2	2397	861					
29d	4	mc2	c2	2313	482					
29d	4	mc3	c2	2150	855					
29d	4	mc4	c2	2159	775					
29d	4	mc1	l1	2150	992					
29d	4	mc2	l1	1926	1040					
29d	4	mc3	l1	2458	989					
29d	4	mc4	l1	2342	306					
29d	4	mc1	l2	2151	1075					
29d	4	mc2	l2	1881	1069					
29d	4	mc3	l2	2309	752					
29d	4	mc4	l2	2442	934					
29d	5	mc1	c1	2294	739		collar	2138.13	459.88	0.22
29d	5	mc2	c1	2129	652		lip	1778.25	650.25	0.37
29d	5	mc3	c1	2180	641					
29d	5	mc4	c1	1948	275					
29d	5	mc1	c2	2279	244					
29d	5	mc2	c2	2130	449					
29d	5	mc3	c2	2011	525					
29d	5	mc4	c2	2134	154					
29d	5	mc1	l1	2055	879					
29d	5	mc2	l1	1797	564					
29d	5	mc3	l1	2137	370					
29d	5	mc4	l1	1815	630					
29d	5	mc1	l2	1724	791					
29d	5	mc2	l2	1459	945					
29d	5	mc3	l2	1461	474					
29d	5	mc4	l2	1778	549					
29d	6	mc1	c1	2266	361		collar	2023.25	647.75	0.32
29d	6	mc2	c1	2127	607		lip	1716.38	782.13	0.46
29d	6	mc3	c1	1961	908					
29d	6	mc4	c1	2048	450					
29d	6	mc1	c2	2077	870					
29d	6	mc2	c2	2055	601					
29d	6	mc3	c2	1946	810					
29d	6	mc4	c2	1706	575					
29d	6	mc1	l1	1966	783					

29d	6	mc2	l1	1570	790					
29d	6	mc3	l1	1461	727					
29d	6	mc4	l1	1148	866					
29d	6	mc1	l2	1716	893					
29d	6	mc2	l2	1800	412					
29d	6	mc3	l2	1767	775					
29d	6	mc4	l2	2303	1011					
29d	7	mc1	c1	2389	565		collar	2289.75	731.50	0.32
29d	7	mc2	c1	2305	1080		lip	2341.00	757.88	0.32
29d	7	mc3	c1	2391	272					
29d	7	mc4	c1	1808	937					
29d	7	mc1	c2	2584	406					
29d	7	mc2	c2	2464	399					
29d	7	mc3	c2	2506	1163					
29d	7	mc4	c2	1871	1030					
29d	7	mc1	l1	2251	511					
29d	7	mc2	l1	2144	659					
29d	7	mc3	l1	2125	1063					
29d	7	mc4	l1	2555	564					
29d	7	mc1	l2	2261	634					
29d	7	mc2	l2	2426	961					
29d	7	mc3	l2	2381	1088					
29d	7	mc4	l2	2585	583					
29d	8	mc1	c1	2080	984		collar	2232.63	816.63	0.37
29d	8	mc2	c1	2287	806		lip	2193.88	775.38	0.35
29d	8	mc3	c1	2442	833					
29d	8	mc4	c1	2071	984					
29d	8	mc1	c2	2326	868					
29d	8	mc2	c2	2259	857					
29d	8	mc3	c2	2282	551					
29d	8	mc4	c2	2114	650					
29d	8	mc1	l1	1961	1016					
29d	8	mc2	l1	2566	582					
29d	8	mc3	l1	1808	813					
29d	8	mc4	l1	2417	770					
29d	8	mc1	l2	2182	847					
29d	8	mc2	l2	2266	963					

29d	8	mc3	l2	2088	1050					
29d	8	mc4	l2	2263	162					
29d	9	mc1	c1	2427	481		collar	2424.38	721.88	0.30
29d	9	mc2	c1	2351	967		lip	2119.88	927.50	0.44
29d	9	mc3	c1	2437	1070					
29d	9	mc4	c1	2527	788					
29d	9	mc1	c2	2370	176					
29d	9	mc2	c2	2456	902					
29d	9	mc3	c2	2370	446					
29d	9	mc4	c2	2457	945					
29d	9	mc1	l1	1316	757					
29d	9	mc2	l1	1929	1053					
29d	9	mc3	l1	2195	805					
29d	9	mc4	l1	2217	1119					
29d	9	mc1	l2	2500	701					
29d	9	mc2	l2	2426	1031					
29d	9	mc3	l2	2206	874					
29d	9	mc4	l2	2170	1080					
29d	10	mc1	c1	2368	1153		collar	2396.75	768.88	0.32
29d	10	mc2	c1	2371	798		lip	2026.38	801.75	0.40
29d	10	mc3	c1	2489	1031					
29d	10	mc4	c1	2413	555					
29d	10	mc1	c2	2436	717					
29d	10	mc2	c2	2360	548					
29d	10	mc3	c2	2357	622					
29d	10	mc4	c2	2380	727					
29d	10	mc1	l1	2227	971					
29d	10	mc2	l1	1908	484					
29d	10	mc3	l1	2116	983					
29d	10	mc4	l1	1980	788					
29d	10	mc1	l2	2238	926					
29d	10	mc2	l2	1752	676					
29d	10	mc3	l2	2154	851					
29d	10	mc4	l2	1836	735					
29d	11	mc1	c1	2336	750		collar	2261.63	750.13	0.33
29d	11	mc2	c1	2302	432		lip	2070.75	829.75	0.40
29d	11	mc3	c1	2128	990					

29d	11	mc4	c1	2206	1042					
29d	11	mc1	c2	2433	882					
29d	11	mc2	c2	2341	578					
29d	11	mc3	c2	2311	598					
29d	11	mc4	c2	2036	729					
29d	11	mc1	l1	2222	443					
29d	11	mc2	l1	1766	814					
29d	11	mc3	l1	1860	968					
29d	11	mc4	l1	2104	824					
29d	11	mc1	l2	1837	756					
29d	11	mc2	l2	2271	979					
29d	11	mc3	l2	2369	816					
29d	11	mc4	l2	2137	1038					
29d	12	mc1	c1	2396	519		collar	2339.13	678.13	0.29
29d	12	mc2	c1	2014	974		lip	2229.25	788.88	0.35
29d	12	mc3	c1	2538	527					
29d	12	mc4	c1	2413	901					
29d	12	mc1	c2	2424	702					
29d	12	mc2	c2	2390	439					
29d	12	mc3	c2	2085	530					
29d	12	mc4	c2	2453	833					
29d	12	mc1	l1	1873	648					
29d	12	mc2	l1	2480	713					
29d	12	mc3	l1	2167	900					
29d	12	mc4	l1	2531	1013					
29d	12	mc1	l2	2347	375					
29d	12	mc2	l2	2357	708					
29d	12	mc3	l2	1566	1136					
29d	12	mc4	l2	2513	818					
43d	1	mc1	c1	2045	525		collar	2335.88	817.25	0.35
43d	1	mc2	c1	2405	1143		lip	1975.00	873.75	0.44
43d	1	mc3	c1	2262	960					
43d	1	mc4	c1	2205	952					
43d	1	mc1	c2	2532	799					
43d	1	mc2	c2	2305	814					
43d	1	mc3	c2	2516	1041					
43d	1	mc4	c2	2417	304					

43d	1	mc1	l1	2333	892					
43d	1	mc2	l1	2074	987					
43d	1	mc3	l1	1797	927					
43d	1	mc4	l1	2183	932					
43d	1	mc1	l2	1768	730					
43d	1	mc2	l2	1972	746					
43d	1	mc3	l2	1643	825					
43d	1	mc4	l2	2030	951					
43d	2	mc1	c1	2197	610		collar	2301.00	706.88	0.31
43d	2	mc2	c1	2275	303		lip	2009.13	910.25	0.45
43d	2	mc3	c1	1940	1043					
43d	2	mc4	c1	2326	622					
43d	2	mc1	c2	2497	362					
43d	2	mc2	c2	2314	935					
43d	2	mc3	c2	2361	750					
43d	2	mc4	c2	2498	1030					
43d	2	mc1	l1	1661	628					
43d	2	mc2	l1	1863	925					
43d	2	mc3	l1	1604	1027					
43d	2	mc4	l1	2394	1027					
43d	2	mc1	l2	1842	556					
43d	2	mc2	l2	1857	1204					
43d	2	mc3	l2	2680	997					
43d	2	mc4	l2	2172	918					
43d	3	mc1	c1	1881	784		collar	2077.13	774.75	0.37
43d	3	mc2	c1	1963	619		lip	1792.88	819.63	0.46
43d	3	mc3	c1	1828	998					
43d	3	mc4	c1	2098	731					
43d	3	mc1	c2	2179	620					
43d	3	mc2	c2	2145	962					
43d	3	mc3	c2	2286	955					
43d	3	mc4	c2	2237	529					
43d	3	mc1	l1	1834	782					
43d	3	mc2	l1	2273	881					
43d	3	mc3	l1	2033	849					
43d	3	mc4	l1	2125	1032					
43d	3	mc1	l2	1091	597					

43d	3	mc2	l2	1569	937					
43d	3	mc3	l2	1645	608					
43d	3	mc4	l2	1773	871					
43d	4	mc1	c1	2211	1076		collar	2270.50	822.88	0.36
43d	4	mc2	c1	2502	1074		lip	2008.38	935.00	0.47
43d	4	mc3	c1	2448	275					
43d	4	mc4	c1	2369	616					
43d	4	mc1	c2	2250	763					
43d	4	mc2	c2	2077	747					
43d	4	mc3	c2	2126	1086					
43d	4	mc4	c2	2181	946					
43d	4	mc1	l1	1992	704					
43d	4	mc2	l1	1775	1106					
43d	4	mc3	l1	2065	1008					
43d	4	mc4	l1	2252	913					
43d	4	mc1	l2	1854	966					
43d	4	mc2	l2	1927	1014					
43d	4	mc3	l2	2021	945					
43d	4	mc4	l2	2181	824					
43d	5	mc1	c1	1956	767		collar	2137.88	678.50	0.32
43d	5	mc2	c1	2018	1090		lip	1420.00	640.25	0.45
43d	5	mc3	c1	1984	588					
43d	5	mc4	c1	2361	253					
43d	5	mc1	c2	2159	610					
43d	5	mc2	c2	2333	469					
43d	5	mc3	c2	2141	962					
43d	5	mc4	c2	2151	689					
43d	5	mc1	l1	1211	477					
43d	5	mc2	l1	1801	761					
43d	5	mc3	l1	1277	875					
43d	5	mc4	l1	1275	644					
43d	5	mc1	l2	1505	619					
43d	5	mc2	l2	1360	961					
43d	5	mc3	l2	1086	194					
43d	5	mc4	l2	1845	591					
43d	6	mc1	c1	2440	1177		collar	2266.88	748.63	0.33
43d	6	mc2	c1	2351	940		lip	1969.75	955.00	0.48

43d	6	mc3	c1	2325	830					
43d	6	mc4	c1	1887	444					
43d	6	mc1	c2	2298	541					
43d	6	mc2	c2	2201	927					
43d	6	mc3	c2	2382	897					
43d	6	mc4	c2	2251	233					
43d	6	mc1	l1	2055	686					
43d	6	mc2	l1	2471	822					
43d	6	mc3	l1	1693	725					
43d	6	mc4	l1	1823	1100					
43d	6	mc1	l2	2294	821					
43d	6	mc2	l2	2198	998					
43d	6	mc3	l2	1260	1176					
43d	6	mc4	l2	1964	1312					
43d	7	mc1	c1	1995	956		collar	2178.88	640.25	0.29
43d	7	mc2	c1	2093	202		lip	2033.00	622.00	0.31
43d	7	mc3	c1	2348	1048					
43d	7	mc4	c1	2388	119					
43d	7	mc1	c2	2021	1280					
43d	7	mc2	c2	2005	717					
43d	7	mc3	c2	2275	395					
43d	7	mc4	c2	2306	405					
43d	7	mc1	l1	2276	1013					
43d	7	mc2	l1	2257	942					
43d	7	mc3	l1	2081	637					
43d	7	mc4	l1	2317	645					
43d	7	mc1	l2	1484	273					
43d	7	mc2	l2	2172	403					
43d	7	mc3	l2	1676	746					
43d	7	mc4	l2	2001	317					
43d	8	mc1	c1	1735	366		collar	2244.00	806.63	0.36
43d	8	mc2	c1	2461	994		lip	1992.25	836.75	0.42
43d	8	mc3	c1	2304	942					
43d	8	mc4	c1	2217	956					
43d	8	mc1	c2	2418	899					
43d	8	mc2	c2	2430	837					
43d	8	mc3	c2	2128	755					

43d	8	mc4	c2	2259	704					
43d	8	mc1	l1	1610	520					
43d	8	mc2	l1	1962	885					
43d	8	mc3	l1	2290	1074					
43d	8	mc4	l1	2278	738					
43d	8	mc1	l2	2725	586					
43d	8	mc2	l2	1846	1096					
43d	8	mc3	l2	2387	977					
43d	8	mc4	l2	840	818					
43d	9	mc1	c1	1862	751		collar	2138.25	790.75	0.37
43d	9	mc2	c1	1926	821		lip	1810.63	845.75	0.47
43d	9	mc3	c1	2366	1060					
43d	9	mc4	c1	2057	636					
43d	9	mc1	c2	2083	426					
43d	9	mc2	c2	2092	936					
43d	9	mc3	c2	2477	1081					
43d	9	mc4	c2	2243	615					
43d	9	mc1	l1	1178	818					
43d	9	mc2	l1	1773	722					
43d	9	mc3	l1	2054	901					
43d	9	mc4	l1	1416	1041					
43d	9	mc1	l2	2255	983					
43d	9	mc2	l2	1639	952					
43d	9	mc3	l2	2083	347					
43d	9	mc4	l2	2087	1002					
43d	10	mc1	c1	2132	1078		collar	2333.38	896.63	0.38
43d	10	mc2	c1	2426	1057		lip	2038.25	1006.63	0.49
43d	10	mc3	c1	2651	1021					
43d	10	mc4	c1	2480	674					
43d	10	mc1	c2	2378	709					
43d	10	mc2	c2	2114	918					
43d	10	mc3	c2	2310	913					
43d	10	mc4	c2	2176	803					
43d	10	mc1	l1	2322	1183					
43d	10	mc2	l1	2045	959					
43d	10	mc3	l1	2023	894					
43d	10	mc4	l1	1906	822					

43d	10	mc1	l2	2257	1063					
43d	10	mc2	l2	1846	783					
43d	10	mc3	l2	2367	1121					
43d	10	mc4	l2	1540	1228					
43d	11	mc1	c1	2160	1101		collar	2358.25	837.00	0.35
43d	11	mc2	c1	2418	999		lip	2287.75	1065.88	0.47
43d	11	mc3	c1	2183	1164					
43d	11	mc4	c1	2229	654					
43d	11	mc1	c2	2403	492					
43d	11	mc2	c2	2475	428					
43d	11	mc3	c2	2503	805					
43d	11	mc4	c2	2495	1053					
43d	11	mc1	l1	2396	1088					
43d	11	mc2	l1	2368	842					
43d	11	mc3	l1	2602	1264					
43d	11	mc4	l1	2389	1158					
43d	11	mc1	l2	2550	838					
43d	11	mc2	l2	1410	1267					
43d	11	mc3	l2	1976	1159					
43d	11	mc4	l2	2611	911					
43d	12	mc1	c1	2360	1070		collar	2171.38	889.63	0.41
43d	12	mc2	c1	2153	789		lip	2081.75	891.00	0.43
43d	12	mc3	c1	2150	883					
43d	12	mc4	c1	2133	559					
43d	12	mc1	c2	2234	922					
43d	12	mc2	c2	2019	854					
43d	12	mc3	c2	2100	1069					
43d	12	mc4	c2	2222	971					
43d	12	mc1	l1	2129	968					
43d	12	mc2	l1	2465	912					
43d	12	mc3	l1	1593	788					
43d	12	mc4	l1	2074	686					
43d	12	mc1	l2	2196	918					
43d	12	mc2	l2	2132	844					
43d	12	mc3	l2	2002	1169					
43d	12	mc4	l2	2063	843					
